# Protocol of the study: Multilevel community-based mental health intervention to address structural inequities and adverse disparate consequences of COVID-19 pandemic on Latinx Immigrants and African refugees

**DOI:** 10.1371/journal.pone.0298369

**Published:** 2024-04-16

**Authors:** Jessica R. Goodkind, M. Lee Van Horn, Julia Meredith Hess, David Lardier, Cirila Estela Vasquez Guzman, Janet Ramirez, Susana Echeverri Herrera, Meredith Blackwell, Alejandra Lemus, Bianca Ruiz-Negron, Ryeora Choe

**Affiliations:** 1 Department of Sociology, University of New Mexico, Albuquerque, NM, United States of America; 2 Department of Individual, Family, and Community Education, University of New Mexico, Albuquerque, NM, United States of America; 3 Department of Pediatrics, University of New Mexico, Albuquerque, NM, United States of America; 4 Department of Psychiatry and Behavioral Sciences, University of New Mexico, Albuquerque, NM, United States of America; 5 Department of Family Medicine, Oregon Health & Science University, Portland, Oregon, United States of America; 6 Department of Psychology, University of New Mexico, Albuquerque, NM, United States of America; 7 United Voices for Newcomer Rights, Albuquerque, New Mexico, United States of America; Public Library of Science, UNITED STATES

## Abstract

The NIMH-funded *Multilevel Community-Based Mental Health Intervention to Address Structural Inequities and Adverse Disparate Consequences of COVID-19 Pandemic on Latinx Immigrants and African Refugees* study aims to advance the science of multilevel interventions to reduce the disparate, adverse mental health, behavioral, and socioeconomic consequences of the COVID-19 pandemic that are a result of complex interactions between underlying structural inequities and barriers to health care. The study tests three nested levels of intervention: 1) an efficacious 4-month advocacy and mutual learning model (*Refugee and Immigrant Well-being Project*, *RIWP*); 2) engagement with community-based organizations (CBOs); and 3) structural policy changes enacted in response to the pandemic. This community-based participatory research (CBPR) study builds on long-standing collaboration with five CBOs. By including 240 Latinx immigrants and 60 African refugees recruited from CBO partners who are randomly assigned to treatment-as-usual CBO involvement or the RIWP intervention and a comparison group comprised of a random sample of 300 Latinx immigrants, this mixed methods longitudinal waitlist control group design study with seven time points over 36 months tests the effectiveness of the RIWP intervention and engagement with CBOs to reduce psychological distress, daily stressors, and economic precarity and increase protective factors (social support, access to resources, English proficiency, cultural connectedness). The study also tests the ability of the RIWP intervention and engagement with CBOs to increase access to the direct benefits of structural interventions. This paper reports on the theoretical basis, design, qualitative and quantitative analysis plan, and power for the study.

## Introduction

### Disparate impact of COVID-19 on Latinx and African newcomers

The immigrant share of the U.S. population comprised 13.6% of the total population in 2021 (27% when including U.S.-born children of immigrants) [1], with Latinx immigrants accounting for 44% of all immigrants and Africans comprising 5.7% [2]. Due to long-standing structural inequities, the COVID-19 pandemic has disparately impacted immigrants and refugees, particularly those who are Latinx and Black, with Latinx and Black people having age-adjusted mortality rates of 510 and 489 deaths per 100,000 respectively compared to 311 for non-Hispanic Whites [3]. Latinx and Black populations have also experienced disparities in economic impact, including food and housing insecurity, with many Latinx immigrants having limited access to government economic relief [4]. These disparities are related to multiple factors, including the large percentage of essential workers among Latinx and Black populations, language barriers, multi-generational homes, racism, immigration status that limits access to health care, lack of transportation, and disparities in access to paid leave and health insurance [5, 6]. Mental health consequences of the pandemic have also been severe, with disparate impacts rooted in structural inequities operating through pathways including increased exposure to grief, economic insecurity, frontline healthcare work, and poor physical health [7].

### Mental health and access to care among Latinx and African newcomers

The social, political, legal, and economic context of uncertainty, stigma, fear of accessing health care and other resources, detention, deportation, and family separation based on current immigration policies and the broader public perception of immigrants as a threat all play a critical role in the mental health of immigrants in the U.S. [8, 9]. Similarly, refugees often have high rates of PTSD, depression, and other forms of psychological distress, limited material resources, lingering physical ailments, and loss of meaningful social roles and support, all of which are compounded by structural inequities and marginalization of their cultural practices [10, 11]. Although there are differences between refugees and immigrants in terms of legal status, most migrants leave their home countries due to an intersection of “push” and “pull” factors (including trauma exposure in their home country, during flight, and in reception/ resettlement contexts). Recognizing commonalities in past experiences and resettlement challenges, we use the inclusive, non-stigmatized term newcomers to encompass immigrants, refugees, and asylum seekers. Despite higher rates of psychological distress, newcomers have low utilization rates of mental health services, in part due to barriers of lack of health insurance or eligibility for government health programs, stigma, lack of interpretation services and culturally appropriate care, and policies such as the Public Charge Rule that put immigrants’ ability to stay in the U.S. at risk if they accessed government benefits [12].

Although evidence points to the need to address socio-structural determinants, many mental health interventions offered to newcomers focus on individual-level predictors of mental health [13, 14]. Also, Latinx immigrant health outcomes are often viewed within the Hispanic Health Paradox, which suggests that Latinx immigrants have better health than non-Hispanic whites in the U.S. [15], and therefore are frequently overlooked in mental health research and development of interventions [16], despite mounting evidence of mental health disparities, structural inequities, and disproportionate exposure to trauma [17, 18]. The pandemic has impacted the mental health of newcomers in particular ways because some of its impacts (death, isolation, confinement to home, exposure risks as essential workers) can be re-traumatizing, have increased immigration-related uncertainty and socioeconomic precarity (pay cuts, job loss, health care access, immigration status), and exacerbated family separation because of increased restrictions on movement [19, 20]. However, for some newcomers, the pandemic and its consequences are not as unfamiliar because of past experiences with crises. Thus, strengths on which to build are newcomers’ coping strategies for managing crises and abilities to overcome adversity [21]. In sum, the starkly disparate health and socioeconomic consequences of the COVID-19 pandemic on Latinx and Black newcomers have highlighted the urgent necessity for community-based, multilevel, intervention approaches that address social-structural determinants, are culturally appropriate, strengths-based, scalable, and occur in non-stigmatized settings.

### Study design, aims, and hypotheses

**Fig 1:**
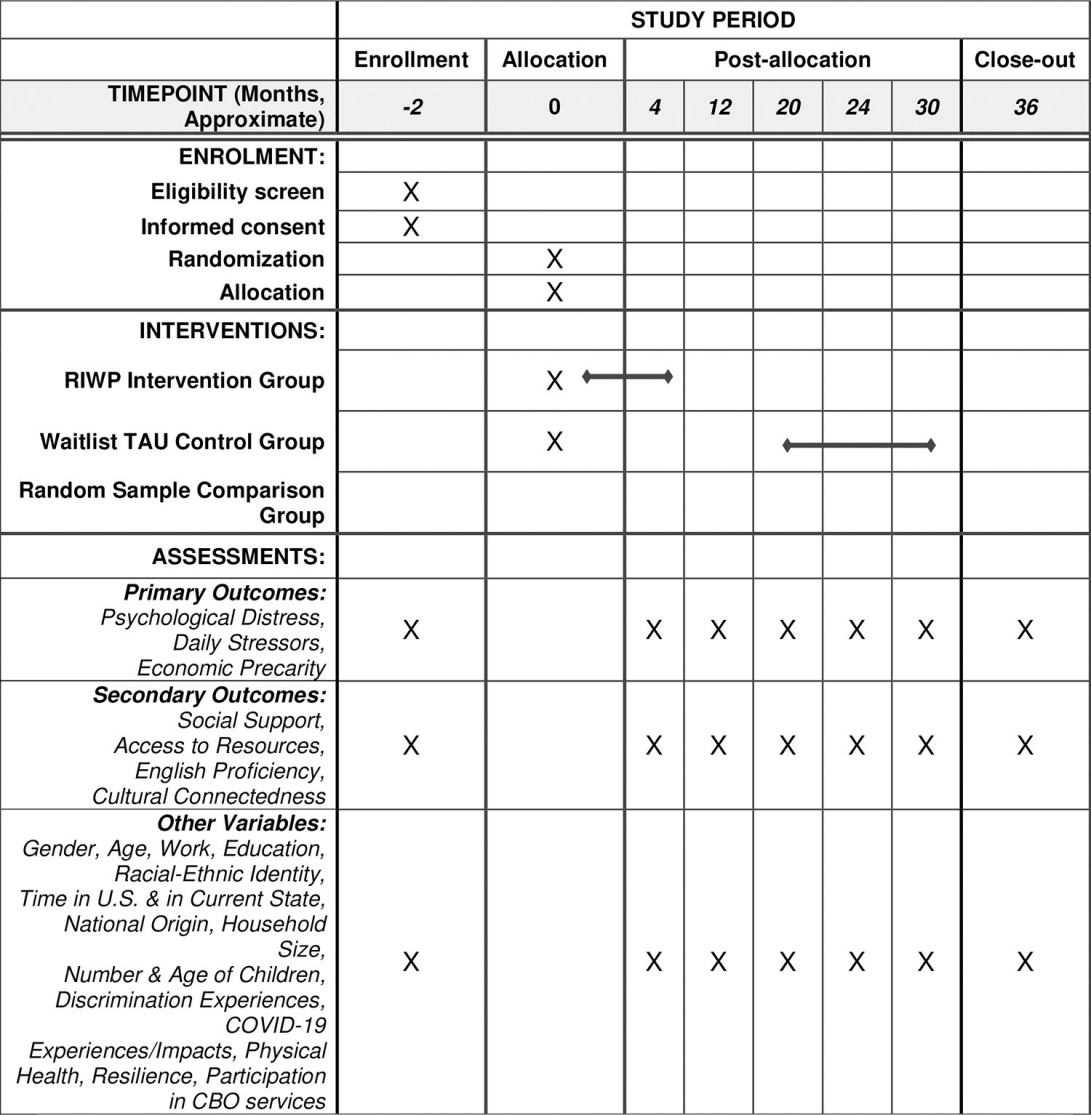
Schedule of enrolment, interventions, and assessments. Time points are approximate; all assessments typically take 4 months to complete. Outcome and other variables collected at all timepoints, including baseline. CBO = community-based organization.

**Fig 2:**
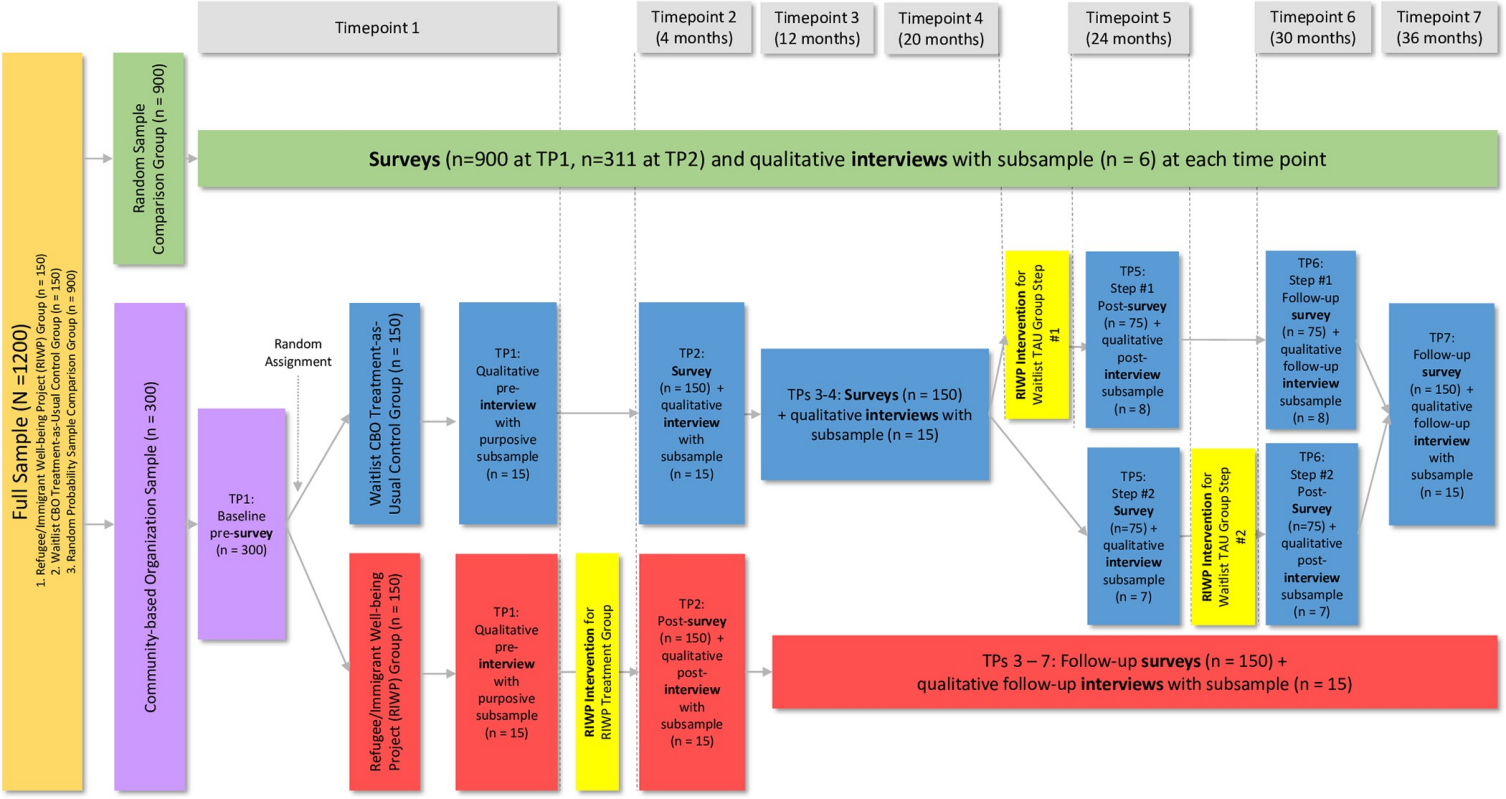
Research design/consort diagram.

This paper reports the protocol for the NIMH funded *Multilevel Community-Based Mental Health Intervention to Address Structural Inequities and Adverse Disparate Consequences of COVID-19 Pandemic on Latinx Immigrants and African Refugees* study, which is evaluating the ability of the RIWP intervention and community-based organizations (CBOs) to reduce adverse consequences of the COVID-19 pandemic for Latinx and African newcomers. This longitudinal, mixed-methods, waitlist control group design study with 7 timepoints of data collection over 36 months includes a randomized test of the RIWP intervention, a community comparison for the evaluation of the role of CBOs, and a qualitative assessment of mechanisms of change, the effects of policy interventions on individuals, how CBOs contribute to enacting policies and helping people benefit from them, and the context of RIWP implementation at each site (see Figs 1 and 2 for SPIRIT Schedule and Research Design/Consort Diagram and S1Checklist for SPIRIT Checklist). The primary aims of the study are to:

1. Test the effects of the RIWP intervention to reduce adverse consequences of the COVID-19 pandemic for Latinx and African newcomers. The primary hypothesis is that RIWP participants will have decreased psychological distress, daily stressors, and economic precarity as compared to randomly assigned treatment-as-usual waitlist control group. The secondary hypothesis is that RIWP participants will have increased protective factors (social support, access to resources, English proficiency, cultural connectedness).
2. Test the effects of engagement with CBOs to reduce adverse consequences of the COVID-19 pandemic for Latinx and Black newcomer. The primary hypothesis is that CBO participants will have decreased psychological distress, daily stressors, and economic precarity as compared to the comparison group of randomly sampled immigrants. The secondary hypothesis is that CBO participants will have increased protective factors (social support, access to resources, English proficiency, cultural connectedness).
3. Test the effects of RIWP intervention and engagement with CBOs to increase access to structural interventions (local/state/federal relief) for Latinx and Black newcomers. We hypothesize that CBO and RIWP participants will be more likely to experience benefits of structural policy interventions than the comparison group of randomly sampled newcomers.

## Methods

### Participants

This study has two distinct samples. To test the effects of the RIWP intervention, we are recruiting a sample of Latinx and African immigrants and refugees in a Southwest state in the United States from lists of those served by CBOs with a target of 300 individuals, 60 who are African and 240 who are Latinx. The CBO sample is randomly assigned to receive the RIWP intervention or to a waitlist control condition. For this sample, based on our previous research [22], we expect attrition to be 10% across the course of the study. We note that a small number of participants in this sample, recruited from community events, were not already connected to a CBO; in this case we facilitate a connection to a CBO that serves their community. The second sample consists of 900 Latinx immigrants who live in the same Southwest state recruited by a survey firm specializing in Latinx populations. Respondents are randomly drawn from a mix of phone and web-based data collection, including heavy use of cell-phone numbers. Surveys are conducted through a mixed mode format (web and phone-based interviews). Respondents aged 18 and over who self-identify as Latinx or Hispanic immigrants in this state and are not receiving services from a CBO are eligible. Surveys are conducted by bilingual interviewers. For the phone portion, respondents are greeted in both languages, and surveys are conducted in either English or Spanish, at the discretion of the respondent. Up to five callbacks are scheduled for each record. The same approach is taken for email or text message invites for online surveys. Survey firm software allows tight control of the sample by tracking call histories for every number dialed and every email/text invitation, to ensure that times when respondents are contacted vary to maximize response rates. This sample is used for the comparison group for aims 2 and 3 and is not offered the RIWP intervention or randomly assigned to treatment. This sample size was increased from the originally planned 300 because of concerns that attrition would be greater in this random sample without ties to CBOs. Compensation for participants’ time for each survey starts at $25 and increases as follows: T1 $25, T2 $40, T3 $50, T4-T7 $60. Participants provide verbal consent, which is witnessed by the person conducting the survey and documented by the participant’s willingness to answer the survey questions. A subset of 36 participants are selected from the three samples for qualitative interviews. This sample is selected to include 30 from the CBO sample [15 from the RIWP intervention group (12 Latinx, 3 African) and 15 from the waitlist control group (12 Latinx, 3 African)], and 6 from the random sample comparison group and to be balanced on gender. Participants are offered an additional $25 for their time.

### Community-based organizations

CBOs are an important part of the intervention, which ties into existing systems of care and involves helping newcomers utilize available resources to meet their needs. CBO partners provide a range of resources including legal, education, housing, health care, and community mobilization. CBOs each receive $30,000 for each year of the study to participate in all aspects of the research, including design, participant recruitment, implementation, data analysis, and dissemination. By recruiting from CBOs, the study utilizes existing ties to increase engagement and to provide the opportunity to test the effects of CBO engagement on mental health, stressors, and economic precarity. Initially we planned to work with 5 CBOs who were partners in a previous study of the RIWP intervention to recruit from their list of participants. However, in the course of recruitment, it became clear that one of the impacts of the pandemic was that CBOs lost contact with many of their participants and had staffing challenges (e.g., staff were reduced, worked remotely) that made it difficult to obtain our goal number of participants. We addressed this both by adding an additional 8 CBOs and by recruiting from community events.

### Retention strategies

In our previous RCT of RIWP, we retained 100% of 143 households over 14 months and 4 time points of data collection, with all 290 participants completing at least 2 interviews. These high retention rates are due to numerous strategies, which will be employed in this study:

- Community-based participatory research (CBPR) approach
- Depth of our community engagement with refugee and immigrant leaders and community-based organizations (CBOs)
- Cultural appropriateness of the survey and interview questions
- Cultural concordance of study team members
- Collection of multiple ways to contact each participant (e.g., phone numbers of at least 3 family members/friends, multi-modal contact methods including phone, email, address)
- Careful tracking of all contacts and attempted contacts in database, with details about challenges/issues
- Comprehensive retention protocol for longitudinal research that involves periodic phone, mail, and email contact and accessing participants’ social and community networks
- Increasing compensation for each interview time point

### Timeline

This study was approved by the University of New Mexico IRB (see S1 Protocol for IRB-approved protocol) and funded in the summer of 2021 and released in ClinicalTrials.gov (NCT05092542) on October 22, 2021. Participant recruitment and the first data collection point occurred between October 19, 2021, and May 4, 2022, which included the Omicron wave of the COVID-19 pandemic (the highest rate of COVID infections in the state was during this time period) and widespread availability of COVID vaccine boosters. At the start of data collection, 62.2% of New Mexicans had received a full dose of the COVID vaccine; by the end of the first timepoint, these numbers increased to 70.4% being fully vaccinated and 86.8% having 1 dose [6]. The second wave of data collection is 4 months after the first wave; subsequent waves are every 4–8 months for 3 years from baseline to final data collection. We allow for a fairly wide window for each of these data collection points, which means that there is close to continuous coverage of outcomes across the study. Those on the waitlist will be offered participation in the full intervention in either the fall of 2023 or spring of 2024. Inspired by a step-wedge design, the waitlist control individuals receive the intervention in two different waves (either after the 4^th^ or 5^th^ assessment).

### Randomization

All participants in the CBO sample are randomly assigned after completing the baseline assessment and agreeing that they could be in a particular RIWP intervention group time, if assigned. Assignment to waitlist control or the treatment condition is made with a probability of .5, blocking on CBO to assure balance across each organization. Because each intervention group starts once a particular time slot fills, recruitment is ongoing over six months. Separate confidential lists for each CBO of treatment assignment (with treatment randomly assigned for every two participants) were generated by the study statistician with participants assigned after baseline assessment. Once 15 treatment participants are selected for a given intervention group, then that group is designated as full. This procedure assures that each intervention group has its own control group that is balanced in terms of time, availability to participate, and CBO.

### Intervention

#### Level 1: RIWP intervention

**Fig 3:**
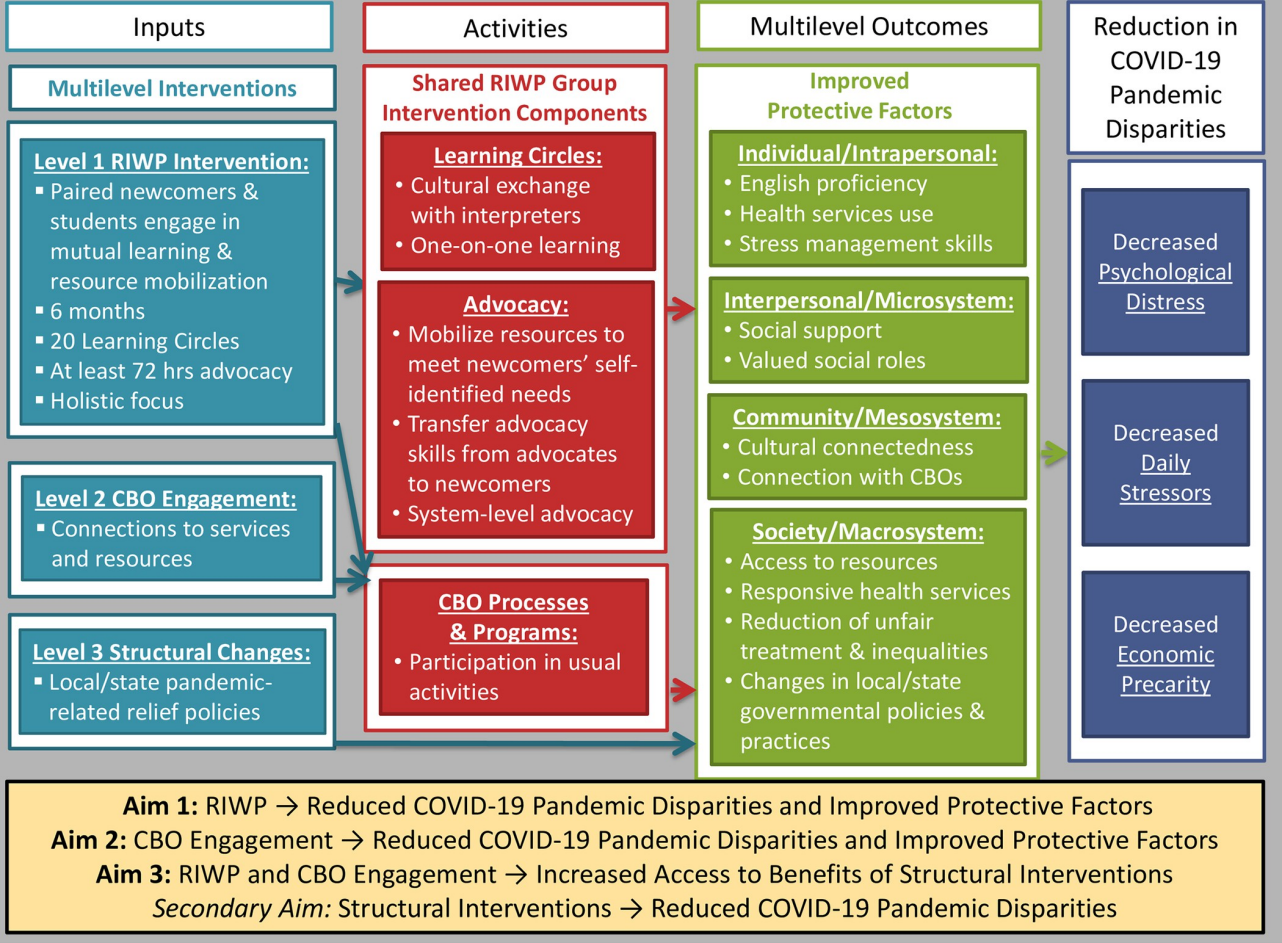
Conceptual model of multiple levels of intervention.

RIWP involves a sustainable and replicable partnership model between refugees, CBOs, and universities (see Fig 3 for Conceptual Model). Newcomer families and undergraduate advocates work together for 4 months to: a) increase newcomers’ abilities to navigate their new communities; b) improve newcomers’ access to community resources; c) enhance meaningful social roles by valuing newcomers’ cultures, experiences, and knowledge; d) reduce newcomers’ social isolation; and e) increase community responsiveness to newcomers. RIWP is implemented by university students enrolled in a 2-semester course, and has two elements: 1) Learning Circles, which involve cultural exchange and one-on-one learning opportunities; and 2) Advocacy, which involve collaborative efforts to mobilize community resources related to health, housing, employment, education, and legal issues and to create policy/system changes. Meeting in weekly Learning Circles, newcomers and their student partners learn from each other during cultural exchange time designed to facilitate sharing of knowledge and to help newcomers recognize their potential to effect changes in their communities. During one-on-one learning time, newcomers and students practice English, fill out job applications, and engage in other activities newcomers want to pursue to expand knowledge, improve skills, and accomplish goals.

RIWP is holistic, multilevel, and strengths-based, and has an explicit social justice orientation that is informed by an ecological perspective. This holistic intervention is designed to addresses the multiple sources of newcomers’ distress, including psychological (past traumas), material (lack of access to resources), physical (physical ailments from violence and deprivation), social (loss of meaningful social roles and social support), educational (limited English proficiency), and cultural (disconnection from traditional cultural practices). A strengths-based perspective is important because newcomers have survived in the face of tremendous hardships and have numerous strengths on which to build, including cultural knowledge, resourcefulness, effective coping strategies, multilingual abilities, and often strong family and community bonds and support. Thus, mutual learning is an intentional and core component of RIWP. This emphasis also ensures that RIWP is appropriate for multiple linguistic and cultural groups who can participate simultaneously and learn from one another as well. Ensuring that RIWP creates change at multiple levels is important because newcomers have urgent learning, resource, and health needs that must be addressed at the individual level in order to enable them to work collectively with others to create community and structural changes that address the root causes of mental health disparities and social inequities. A series of studies, including a large RCT, have built strong evidence of the feasibility, acceptability, and effectiveness of the RIWP intervention [22–28].

Trained undergraduates are involved in RIWP and receive course credit. Undergrads make a 2-semester commitment and learn about newcomer mental health, adult education and social change, the experiences of immigrants and refugees, and social determinants of mental health. Undergrads also learn to identify and make connections with community resources, as well as empathy, values clarification and problem-solving skills. Weekly supervision replaces training after the intervention begins. To ensure that all undergrads provide high quality intervention, we employ a careful application and screening process, in-depth training and assessment of skills, and close supervision. We also monitor undergrads’ performance through independent verification with participants. A strength of RIWP is that it utilizes the natural resource of undergrads who need meaningful learning opportunities and who have time through the structure of the course to develop relationships that facilitate transformative change.

#### Level 2: CBO engagement

Our Stage I adaptation of RIWP focused on integrating the intervention into efforts of existing CBOs that are rooted in newcomer communities, and findings highlight the importance of CBOs in RIWP implementation. For example, CBO staff served as Learning Circle facilitators and interpreters and were instrumental in student advocacy efforts because of their 1) in-depth knowledge of community resources and how to navigate them, including but not limited to the services and resources offered at each of their organizations, and 2) lived experience and/or understanding of newcomer experiences through their work at CBOs [29]. In addition, CBOs have been instrumental in providing social and economic support during the pandemic, ensuring that newcomers benefit from pandemic-related resources and building solidarity between refugees and immigrants. This is consistent with other research documenting the important roles CBOs play in the lives of newcomers in terms of increasing access to resources [30], cultural connectedness and capacity building [31], and buffering the effects of migration losses and discrimination [32]. However, little is known about the effects of CBOs organizing across social sectors and diverse cultural groups to reduce pandemic-related disparities experienced by newcomers.

#### Level 3: Structural changes

The COVID-19 pandemic provides a unique opportunity to look at changes in macro-level policies and assistance as local, state, and federal funding was available to support many policies aimed at reducing the negative impacts of the pandemic. Federal policies such as the child tax credit benefited many newcomers but excluded people without a social security number (even if they were taxpayers). The state where the study took place implemented several policies that were either available to newcomers or specifically for them (e.g., a relief bill utilizing the state’s unspent federal aid to provide an additional $1,200 in economic stimulus payments for all unemployed workers who were ineligible for a federal stimulus check). Other state policy efforts included extending eligibility for health coverage to some immigrants, making up for the lack of eligibility among many immigrants for federal supports through the Healthy Workplaces Act to support paid sick leave, and the extension of the working families tax credit to undocumented workers. In addition, local relief policies in the region focused on rental assistance, with emphasis on providing support to people who were not eligible for federal relief.

It is critical to study the impact of policies because individual and family-level stressors that newcomers face are inextricability linked to the socio-structural-political environment [33, 34]. Interventions that incorporate the broad and multilevel nature of the experiences that newcomers face before, during, and post-migration and the structural determinants that impact their mental health outcomes are essential [35]. Rigorous research that directly links policy changes to individual outcomes and that elucidates the effects of CBO engagement and the RIWP intervention on access to benefits of policy changes is a critical next step in improving our understanding of the complex, multilevel processes needed to reduce structural inequities and the health disparities that result.

### Qualitative interview guide

The semi-structured qualitative interview guide was developed by the research team, including CBO members. The guide includes 32 open-ended questions that ask about work, education, migration experience, post-resettlement experiences with discrimination and racism, belonging and feeling valued, and COVID-19 impacts. Participants are asked in-depth about well-being, stress, recovery, help-seeking, and access to care, community participation, and connections to CBOs and impacts. Interview guides for following timepoints include questions for those participating in the RIWP and waitlist control groups about their experiences with CBOs and, if applicable, the RIWP intervention and changes they attribute to their participation. The interview guide was written in Spanish and translated to English. It was then translated and back translated in Swahili and Kinyarwanda/ Kirundi. Interviews with Latinx participants are conducted in Spanish; interviews with Africans are conducted in English with interpreters.

### Quantitative measures

#### Primary outcomes

*Psychological distress*. Psychological distress is assessed using the *Patient Health Questionnaire-9* (PHQ-9) [36], the *Generalized Anxiety Disorder Scale* (GAD-7) [37], and the *COVID-19 Mental Health Impacts Scale* [38]. PHQ-9 is a 9-item measure with high reliability, validity, sensitivity, and specificity for major depressive disorder [39]. GAD-7 is a 7-item measure of generalized anxiety disorder that has good reliability, validity, sensitivity, and specificity [37]. We use 5-items from the COVID-19 Mental Health Impacts Scale to assess participants’ distress related to concerns about contracting COVID-19 and not having enough money, food, and sleep because of the pandemic.

*Daily stressors*. Daily stressors are assessed using the *Perceived Stress Scale-4* (PSS) [40]. The PSS-4 is a 4-item self-administered scale used to measure an individual’s level of perceived stress in the past month. As a result, it measures only current (not chronic) levels of perceived stress. Response options range from 0 (Never) to 4 (Very often). The psychometric properties of the PSS demonstrate good internal consistency and reliability across varying population groups both within the United States internationally [41].

*Economic precarity*. Economic precarity is assessed using 2-items from the *RAND American Life Panel Impacts of COVID-19 Survey* about housing and financial stressors related to COVID [42], 2-items from the *Effects of COVID- Outbreak Scale* [43] about economic impacts of the pandemic on the participant their household, and 2 questions about participants’ income and access to health insurance.

#### Secondary outcomes

Secondary outcomes are also assessed at all 7 timepoints, and include: **Social Support (***Modified Medical Outcomes Study Social Support Survey*) [44], **Access to Resources** (*Satisfaction with Resources Scale* [45], **English Proficiency** (4-items from the *Immigration Policy Lab Integration Index* [46], and **Cultural Connectedness** (modified, parallel versions of the *Language*, *Identity and Behavior Acculturation Scale* [47]. Because connection to one’s native culture and to one’s host country have both been found to be protective for newcomer mental health, we include measures of Home (participant’s native culture) and American cultural connectedness.

#### Covariates

Covariates collected include gender, age, work status, educational background, racial-ethnic identity, time in the United States, time in current state, national origin, household size, number and age of children, household size, discrimination experiences, COVID-19 experiences and impacts, physical health, resilience, and participation in CBO services or programs.

#### Translation of measures

Measures are translated into Swahili, Kirundi/Kinyarwanda, and Spanish using a team approach. Following the Translation, Review, Adjudication, Pretesting, and Documentation process (TRAPD) [48], the research team translates survey measures from English to each of the three languages. Next, these translated measures are given to another team member who translates them back to English. The two English copies are compared, and, if they match appropriately, the translated measures are accepted. If not, the translator, back translator, and other team members meet to discuss discrepancies and reach consensus on translations.

### Qualitative analysis

The qualitative data serve a complementary and explanatory role in conjunction with the primary quantitative design. This project uses a constructivist grounded theory approach to explicate change related to the multiple levels of intervention. Theory is “grounded” in the data themselves. Constructivist grounded theory uses a combined inductive and deductive approach, coupled with the recognition that participants and researchers ‘co-construct’ data [49]. For this CBPR study, qualitative analyses use a team approach. Data analysis is conducted by three related, but progressively larger and more diverse teams. First, a core team comprised of primarily university-based researchers meets weekly to make analysis decisions and continuously analyze incoming data. Second, the larger research team, comprised of the PI, co-investigators, students, staff, and CBO team members meet bi-weekly and provide input on data collection tools, interpretive frameworks, and dissemination products. Third, the study hosts data analysis retreats, which include the entire research team, past research participants and other community members, and additional CBO representatives (see [50] for additional details). To enable participation of CBO and community members in all phases of the analysis, Spanish interviews are transcribed in Spanish and subsequent analysis—coding, queries and memos—are conducted in Spanish or in a bilingual (Spanish/English) approach.

Autocoding by question is the first step in the coding procedure. The team selects thematic clusters that align with the primary research questions of the study and determines which interview questions belong to each theme. Themes may include mental health, access to health care, COVID-19, discrimination, migration, and belonging. Other clusters may be decided upon based on the iterative process of discussing findings. Data analytical processes will follow a similar process for coding the subsequent time points, focusing on change over time in these thematic domains, including how involvement with CBOs and the RIWP intervention impacts participants, their families and communities, and policy change. To examine differences in change over time across treatment groups, we will implement a case study process. Participant interviews are examined for change across time in key areas such as mental health, COVID-19 impacts, intervention effects, and community participation. Team members will write analytic memos to examine the range of experiences, attitudes, commonalities, and differences across particular thematic categories. These multiple processes for focused coding will produce nuanced narratives of multilevel change and how they may differ across treatment groups. These findings will be merged with quantitative findings to explain and expand quantitative findings.

### Quantitative analysis

The longitudinal study design will use random effects (multilevel) models, which account for both repeated measures of individuals and the fact that in one arm individuals are clustered within CBOs. These models allow for outcomes to be missing under the MAR assumption (data is missing at random conditional on the observed outcomes and covariates) [51], which we expect will be adequate. If there is substantial missing data for covariates or evidence that a more inclusive imputation model should be used, then we will use multilevel multiple imputation implemented via the MITML package [52]. Model assumptions will be evaluated using the control group for each analysis and any modification to the analyses to address violations will be made before final analyses using only control group data.

This study is specifically designed to be sensitive to the changing impacts of the pandemic and the effectiveness of multilevel interventions with seven data collection points. Our primary outcomes–psychological distress [53, 54], daily stressors [55, 56], and economic precarity [57, 58] tend to be moderately stable but may also be impacted by shocks such as losing a job or getting COVID-19. Given the unknown trajectory of the pandemic and the path of economic recovery, we do not know the best approach to model the longitudinal data *a priori*. Therefore, we will use the waitlist-control sample and, separately, the representative community sample, to explore longitudinal models which will both capture a large portion of the variability in outcomes over time while also maximizing power to find treatment effects. This allows us to examine possible alternatives in data without treatment effects, resulting in a paper which examines fit of different longitudinal models including additional power analyses before choosing the approach that best balances model fit and power. Analyses assume an intercept and linear change while recognizing that the final longitudinal component may be more complex.

#### Aim 1: Test the effects of the RIWP intervention to reduce adverse consequences of the COVID-19 pandemic

The principal objective of this study is to test the efficacy of the RIWP intervention for participants recruited from CBOs who are randomized to the RIWP intervention or waitlist. We start with a linear model with random effects for individuals. As randomization happens within CBO, there is not an explicit random effect for this included, but we do propose to use sandwich estimators [59, 60] to adjust standard errors for any unexpected impacts of this clustering. In mixed notation, the model for psychological distress is:

$${Distress}_{ti}=B_{00}+{B_{01}(RIWP){+B}_{02}(Time){+B}_{03}(Time*RIWP){+B}_{0N}(covariates)+r_{0ij}+r_{2ij}(Time)+e}_{ij}$$

where participation in the RIWP plus CBO is indicted by a dummy code for RIWP. Because baseline data is collected before randomization, time is centered at baseline and our aim is answered by B_03_ which is the difference in slopes of levels of distress, adjusted for covariates, between those who experience RIWP+CBO intervention and those in the CBO group. Aforementioned covariates will be included in the model. We also include a fixed effect for the Learning Circle time slot (intervention group) they were assigned to.

#### Aim 2. Test the effects of engagement with CBOs to reduce adverse consequences of the COVID-19 pandemic

Analyses for this aim will use both the CBO sample and the random sample of Latinx respondents from across the state. The primary predictor in the analyses is whether the participant is recruited though the CBO or random community sample, and we test whether respondents associated with a CBO differ from those in the random sample in outcomes over time. While the comparison sample is a random sample of Latinx immigrants, the CBO sample consists of respondents already connected to a CBO, thus we propose to use propensity score matching [61, 62] with data collected at baseline to match those in each sample. Our baseline data collection includes variables that are likely to predict an individual’s exposure to a CBO. The model will also adjust for effects of the RIWP intervention observed in aim 1. The adjustment for clustering within CBOs only takes place for those in the CBO arm; in the control arm where there is no nesting, these random effects are assumed to be zero [63, 64]. The model for distress is:

$${Distress}_{tij}=B_{000}+{\gamma_{001}(CBO)+{B_{100}(Time)+\gamma_{101}(CBO*Time)+B}_{010}(covariates)+B_{020}(RIWP)+B_{030}(RIWP*Time)+u_{00j}+u}_{10j}(Time)+r_{0ij}+r_{1ij}(Time)+e_{tij}$$

where CBO is an indicator of whether individual *i* is engaged with the CBO. For this analysis, Time will be centered around the midpoint of the study because engagement with the CBO is ongoing from before the study began. Thus, our aim is answered by γ_001_ which is the difference in mean levels of distress, adjusted for covariates, between those who experience the CBO and those in the random sample and by γ_101_ which is the difference in slopes over time between those who experience the CBO and those in the random sample.

#### Aim 3. Test the effects of RIWP intervention and engagement with CBOs to increase access to benefits of structural interventions

For this aim, the outcome will be a binary indicator for whether each participant accessed the structural interventions that were implemented. The analyses will be similar to Aim 2, but will use a logit link and consequently drop the *e*_*tij*_ term.

#### Secondary aims

As part of the secondary aims analyses, we will use the step-wedge waitlist control design to look at time since treatment started as a predictor of outcomes while also including the dummy variable for initial treatment assignment. The advantage of this approach is that treatment becomes both a between and within participant factor, increasing power and allowing for an assessment of individual differences. Another secondary aim is to examine mechanisms of intervention effectiveness and track local/state/federal policy changes to obtain preliminary estimates of effects of structural interventions on outcomes. This aim will evaluate the impact of receiving the structural interventions which are the outcome of aim 3 on mental health, stressors, and economic precarity. We propose to use propensity scores, but in this case where the treatment is receiving a structural intervention. We will then assess the impact of these interventions using matched samples with random effects models. This is a secondary aim because, while important, there is not enough information to assess power *a priori*. When sufficient information about individual policies is available (date they went into effect, region of effect, eligibility criteria), we will incorporate regression discontinuity analyses [65] to assess treatment effects. An additional aim is to explore whether race/ethnicity, biological sex, gender, sexual orientation, age, SES, and other covariates may moderate intervention impact. For this aim, stratification variables of race/ethnicity, biological sex, gender identity, sexual orientation, age, SES, and intervention setting will be incorporated as level 2 covariates in the models from aim 1. Moderation will be assessed via interactions between the multiple levels of treatment and each of these moderators [66].

#### Power analysis

Power estimates were obtained using a simplified pre/post design, which results in conservative estimates because it does not consider the multiple assessments. For all primary aims, power was calculated using statistical simulations (conducted in R, with analyses run using LME for the continuous outcome and GLMER for the binary outcome), which allow explicit incorporation of the study design. Based on preliminary analyses from similar samples [22], we assume that the correlation over-time between the outcome at baseline and posttest will be .68. We also assume that attrition in the CBO sample will be 10% [22], but we allow for attrition of over 50% in the random sample of Latinx immigrants. Finally, based on the previous trial of the RIWP intervention, observed effect sizes for the significant effects were small to moderate (ranging from Cohen’s D of .30 to .45) [22], thus our objective was to determine the power for an effect size of .3 or more. For aim 1, simulations adjusting for baseline values on the outcome and attrition estimate power to be .91 to detect an effect size of .3. For aims 2 and 3, these analyses assumed random effects for CBO and ICCs of .03 based on previous literature examining outcomes clustered within neighborhoods [67, 68]. For the aim 2 simulations, data were generated according to an ANCOVA model and appropriate type II error control (finding significant effects when there is no effect 5% of the time) was examined by running simulations with an effect size of 0. Across 1000 simulations, we estimate that power to detect an effect size of .30 is .92. For aim 3, power was estimated for a binary outcome with probability of an individual in the control group receiving state level interventions of .2, and the level 2 variance was .10, which is approximately equal to an ICC of .03. We estimated power to detect an odds ratio of 2 for the effect of CBOs on receiving state level of interventions as .82. Power estimates were obtained for secondary aims using power calculators. For secondary aim one, estimates were calculated for a simple ANCOVA using formulas from G*Power [69] and displayed power of .80, with effect size of .23, with power estimates ranging from .70 (d = .19) to .90 (d = .28). For secondary aim two, power was .87, with estimates ranging from .70 (d = .24) to .95 (d = .33). Thus, the study has strong power to detect the hypothesized effects in aim 1 and 2, and moderate power to detect effects for aim 3. Secondary aims also showed moderate to strong power.

### Integration of mixed-methods data

Our study has a complex mixed method design that includes a series of connected projects as reflected in Aims 1–3 [70]. In this mixed method intervention design, the quantitative design is primary and the embedded qualitative strand is a secondary source of data at all timepoints. Quantitative and qualitative data will be integrated in two primary ways. First, the data will be *connected* wherein quantitative data (e.g., demographic information; psychological distress) will be incorporated into our qualitative dataset to inform qualitative data collection from participants. Second, data will be *merged* to explore treatment effects. The qualitative function is to provide convergence, complementarity, and expansion on quantitative data [71]. Convergence occurs when qualitative and quantitative methods are used to answer the same research questions (e.g., how outcomes change over time). Qualitative data will also be used to expand on quantitative findings by providing additional context for significant effects observed and revealing impacts not measured quantitatively.

## Discussion

Building on the growing body of evidence documenting the importance of social-structural determinants of health and mental health for populations disparately impacted by the COVID-19 pandemic, empirical support for the RIWP intervention, and meaningful ongoing community partnerships, this study includes numerous innovations. One is the innovative approach and research design. Our community-based RCT with random assignment of CBO-recruited participants to the RIWP intervention or waitlist CBO treatment-as-usual group, as well as a random sample comparison group, is an innovative and rigorous mixed methods design that builds on a strong community-engaged approach and allows us to test the effects of the RIWP intervention and to test the effects of community and structural level interventions. Through a carefully configured combination of sophisticated quantitative analyses and qualitative data collection, this study examines the direct effects of 3 levels of intervention, moderators of impact (including participants’ age and context of intervention), in addition to measuring multilevel outcomes qualitatively. Thus, this research represents an important next step in the efforts to reduce health disparities in marginalized populations through intervention at multiple levels (individual, interpersonal, community, and society) across multiple domains of influence (behavioral, sociocultural environment, and health care system).

Other key innovations include testing three nested levels of intervention, which offers the unique opportunity to not only examine impacts of structural interventions but also conduct meso-level analyses that explore how individual and CBO interactions intersect to contribute to individuals’ access to benefits of structural interventions. RIWP is integrated into existing efforts at the five partner CBOs, which is premised on the importance of conceptualizing community interventions as complex social processes that aim to create sustainable change at multiple levels. Thus, RIWP is used to leverage and enhance existing services, networks, and community organizing efforts at the CBOs, while also providing the opportunity to separately test the effects of CBO engagement and CBO engagement plus RIWP intervention on addressing the adverse mental health and socioeconomic consequences of the COVID-19 pandemic. Finally, the implementation of RIWP with five CBOs allows for an exploration of different contexts of implementation, and the complex processes within CBOs that lead to sustainable change.

The pandemic has increased the urgency of multilevel change to address structural inequities and resulting disparities in mental health; this study provides a unique opportunity to test the effects of three levels of intervention to reduce adverse consequences of the pandemic that are affecting Latinx and African newcomers (psychological distress, daily stressors, and economic precarity) and increase protective factors (social support, awareness of and access to resources, cultural connectedness, and English proficiency). This is important not only for the large numbers of immigrants and refugees in the U.S. and worldwide, but also to alleviate disproportionate psychological distress experienced by other marginalized populations who experience inequities in social and material resources as well as exposure to trauma and stress, and who are less likely to access formal mental health services because of lack of trust and concerns about culturally appropriateness. In addition, our research aims to elucidate the impacts of individual/interpersonal, community, and societal level interventions/changes on promoting wellbeing and reducing disparities. This study applies an ecological approach that emphasizes recognizing and valuing communities’ histories, strengths, cultures, and social networks, as well as investigating the roles of CBOs in connecting newcomers to resources and promoting structural change. Thus, this study will contribute to the development of the “science of community-level interventions” [72] that examine multiple components of complex social processes and interventions that aim to create sustainable change.

## Supporting information

S1 Checklist SPIRIT 2013 checklist: Recommended items to address in a clinical trial protocol and related documents*. (PDF)

S1 Protocol (DOCX)
