# Supplementary material for: Protocol of the study: Multilevel community-based mental health intervention to address structural inequities and adverse disparate consequences of COVID-19 pandemic on Latinx Immigrants and African refugees
Source: PLoS One. 2024 Apr 16;19(4):e0298369. doi: 10.1371/journal.pone.0298369 (PMC11020834; doi:10.1371/journal.pone.0298369)
Supplement: S1 Protocol — (DOCX) [file pone.0298369.s002.docx]

# UNM IRB PROTOCOL

| TITLE: | Multilevel Community-Based Mental Health Intervention to Address Structural Inequities and Adverse Disparate Consequences of COVID-19 Pandemic on Latinx Immigrants and African Refugees |
| --- | --- |
| VERSION DATE: | 09/28/21 |
| PRINCIPAL INVESTIGATOR/  RESPONSIBLE FACULTY: | Jessica Goodkind |
| STUDENT INVESTIGATOR: | N/A |
| FUNDING AGENCY: | NIH/National Institute of Mental Health(NIMH) – R01MH127733 |

# BACKGROUND/SCIENTIFIC RATIONALE

# The newcomer (immigrant and refugee) share of the U.S. population has risen steadily over the past 50 years, comprising 13.7% of the total population in 2018 (28% of the population when including U.S.-born children of immigrants), with Latinx immigrants accounting for 40% of all immigrants and African immigrants and refugees comprising 5%.^1^ Due to long-standing structural inequities, the COVID-19 pandemic has disparately impacted immigrants and refugees, particularly those who are Latinx, Black, and low-income. Although only 18.5% of the U.S. population is Latinx, 29.5% of COVID-19 cases in which race/ethnicity is known are among the Latinx population. Similarly, Blacks comprise 12.5% of the population but are 30% of COVID-19 cases.^2^ These disparities are compounded by differences in severity and prognosis, with Latinx and Black people 4.2x and 3.9x more likely respectively to be hospitalized for COVID-19 than whites and age-adjusted excess mortality rates of 20.5% and 14.7%.^3^ Other adverse pandemic outcomes include disparities in economic impact (72% of Latinx and 60% of Blacks report serious financial problems versus 36% of whites).^4^ Food and housing insecurity risks are also higher among Latinx (26%, 32% respectively) and Blacks (22%, 28%) than whites (12%, 15%), and many Latinx immigrants have limited access to government economic relief. These disparities are related to multiple factors, including the large percentage of essential workers among the Latinx population (28% of food and agricultural workers) and Black population (22% of transportation, warehouse and delivery workers),^5^ language barriers, multi-generational homes, racism/discrimination, immigration status that may limit access to health care and economic resources, and disparities in access to paid leave and health insurance (uninsured rates among Latinx and Blacks are 2.6x and 1.8x higher than whites). Refugees, particularly those from African countries, have also experienced disparate incidence of COVID-19 and adverse financial and health impacts of the pandemic, due to similar factors that are often further exacerbated by lack of transportation, community integration, and access to television, radio and internet, and underlying pre-migration medical conditions.^6^ Mental health consequences of the pandemic are severe and worsening,^7^ with low-income Latinx and Black populations expected to experience disparate mental health impacts rooted in structural inequities operating through pathways including increased exposure to financial insecurity, grief, frontline healthcare work, and poor physical health.^8^ At the same time, the pandemic has resulted in disruptions in mental health services, particularly those that are community-based.^9^ Despite higher rates of psychological distress, newcomers have low utilization rates of mental health services^18,19^ in part due to barriers of lack of health insurance, ineligibility for government health programs, stigma, lack of interpretation services and culturally appropriate care, and recent policy changes such as the Public Charge Rule that puts immigrants’ ability to stay in the U.S. at risk if they access government benefits.^20–22^ Although evidence points to the need to address socio-structural determinants, many mental health interventions offered to newcomers focus on individual-level predictors of mental health.^23–25^ Also, Latinx immigrant health outcomes are often viewed within the Hispanic Health Paradox (which suggests that Latinx immigrants have better health than non-Hispanic whites in the U.S),^26,27^ and therefore are frequently overlooked in mental health research and development of interventions,^28^ despite mounting evidence of mental health disparities, structural inequities, and disproportionate exposure to trauma.^29–32^ Research has found that the pandemic is impacting the mental health of newcomers in particular ways because some of its impacts (death, crisis, social isolation, and confinement to home, exposure risks as essential workers) can be re-traumatizing, have increased immigration-related uncertainty and fear and socioeconomic precarity (pay cuts, job loss, health care access, immigration status), and exacerbated family separation because of increased restrictions on movement (including resettlement of refugees’ family members). However, for some newcomers, the pandemic and its consequences are not as unfamiliar because of past experiences with crises. Thus, strengths on which to build are newcomers’ coping strategies for managing crisis-related challenges and abilities to overcome adversity.^33^ In sum, the starkly disparate health, mental health, and socioeconomic consequences of the COVID-19 pandemic on Latinx and Black newcomers have highlighted the urgent necessity for community-based, multilevel, multidimensional intervention approaches that address social-structural determinants, are culturally appropriate, strengths-based, cost-effective, and scalable, and occur in non-stigmatized settings.

# OBJECTIVES/AIMS

The goal of this research is to advance the science of multilevel mental health interventions that aim to reduce the disparate, adverse mental health, behavioral, and socioeconomic consequences of the COVID-19 pandemic that are a result of complex interactions between underlying structural inequities and numerous barriers to health care. This community-based participatory research study builds on a long-standing collaboration with 5 community-based organizations (CBOs) and employs a longitudinal waitlist control group design with 5 time points over 32 months. A random probability sample of 1000 Latinx immigrants (RS) will be compared with 240 Latinx immigrants and 60 African refugees recruited through CBOs and randomly assigned to an efficacious peer advocacy and learning model (RIWP; n=150) or a treatment-as-usual waitlist control group (TAU; n=150). This innovative design allows for testing of three nested levels of intervention: 1) peer intervention model; 2) engagement with CBOs; and 3) structural interventions expected in response to the pandemic (e.g., state relief for mixed status families and expanded health insurance coverage), to which all 1300 participants would potentially be exposed. We propose three primary aims to advance mental health intervention and disparities research:

**Aim 1.** Test the effects of the RIWP intervention to reduce adverse consequences of the COVID-19 pandemic for Latinx and Black immigrants and refugees.

**1.1** *Primary Outcomes:* RIWP participants will have decreased psychological distress, daily stressors, and economic precarity as compared to randomly assigned treatment-as-usual waitlist control group (TAU).

**1.2** *Secondary Outcomes:* RIWP participants will have increased protective factors (social support, access to resources, English proficiency, cultural connectedness, health services use).

**Aim 2.** Test the effects of engagement with CBOs to reduce adverse consequences of the COVID-19 pandemic for Latinx and Black immigrants and refugees.

**2.1** *Primary Outcomes:* CBO participants will have decreased psychological distress, daily stressors, and economic precarity as compared to the comparison group of randomly sampled immigrants (RS).

**2.2** *Secondary Outcomes:* CBO participants will have increased protective factors (social support, access to resources, English proficiency, cultural connectedness, health services use).

**Aim 3.** Test the effects of RIWP intervention and engagement with CBOs to increase access to benefits of structural interventions (local/state relief-related policies) for Latinx and Black immigrants and refugees.

**3.1** CBO and RIWP participants will be more likely to experience direct benefits of structural policy interventions than the comparison group of randomly sampled immigrants (RS).

In addition to the primary aims, our study design allows for exploration of key *secondary* aims: a) examine mechanisms of intervention effectiveness; b) test potential moderators of impact; and c) track policy changes to obtain preliminary quantitative estimates of effects of these structural interventions on mental health, stressors, and economic precarity using propensity score matching.^9,10^ Qualitative interviews with a purposive subsample of participants and CBO staff will allow further examination of policy intervention effects, how CBOs contribute to enacting policies and helping people benefit from them, and contexts of RIWP implementation.

Rigorous Experimental Research Design

This mixed methods study will employ a longitudinal randomized waitlist control group design with 5 data collection points over 32 months with 1240 Latinx immigrants and 60 African refugees (see Figure 2). Of this total number, 1000 Latinx immigrants will be a random sample comparison group (we have increased the random sample comparison group from 300 to 1000 participants because we have been advised by our survey consultants/subcontractors that we need to start with 1000 survey participants in the random sample in order to ensure we have an adequate sample size by the 5^th^ timepoint, which is 32 months later). The other 240 Latinx and the 60 African refugees will be recruited through 5 CBO partners, and, in addition to the same timepoints of survey data collection, will be randomly assigned after the first timepoint to the 16-week RIWP intervention or a treatment-as-usual (TAU) waitlist control group (who will receive the RIWP intervention in Year 3). A purposive subsample of 36 participants [15 from RIWP intervention group (12 Latinx and 3 African), 15 from TAU group (12 Latinx and 3 African) and 6 from RS (random sample comparison group)] will complete qualitative interviews at all 5 timepoints.


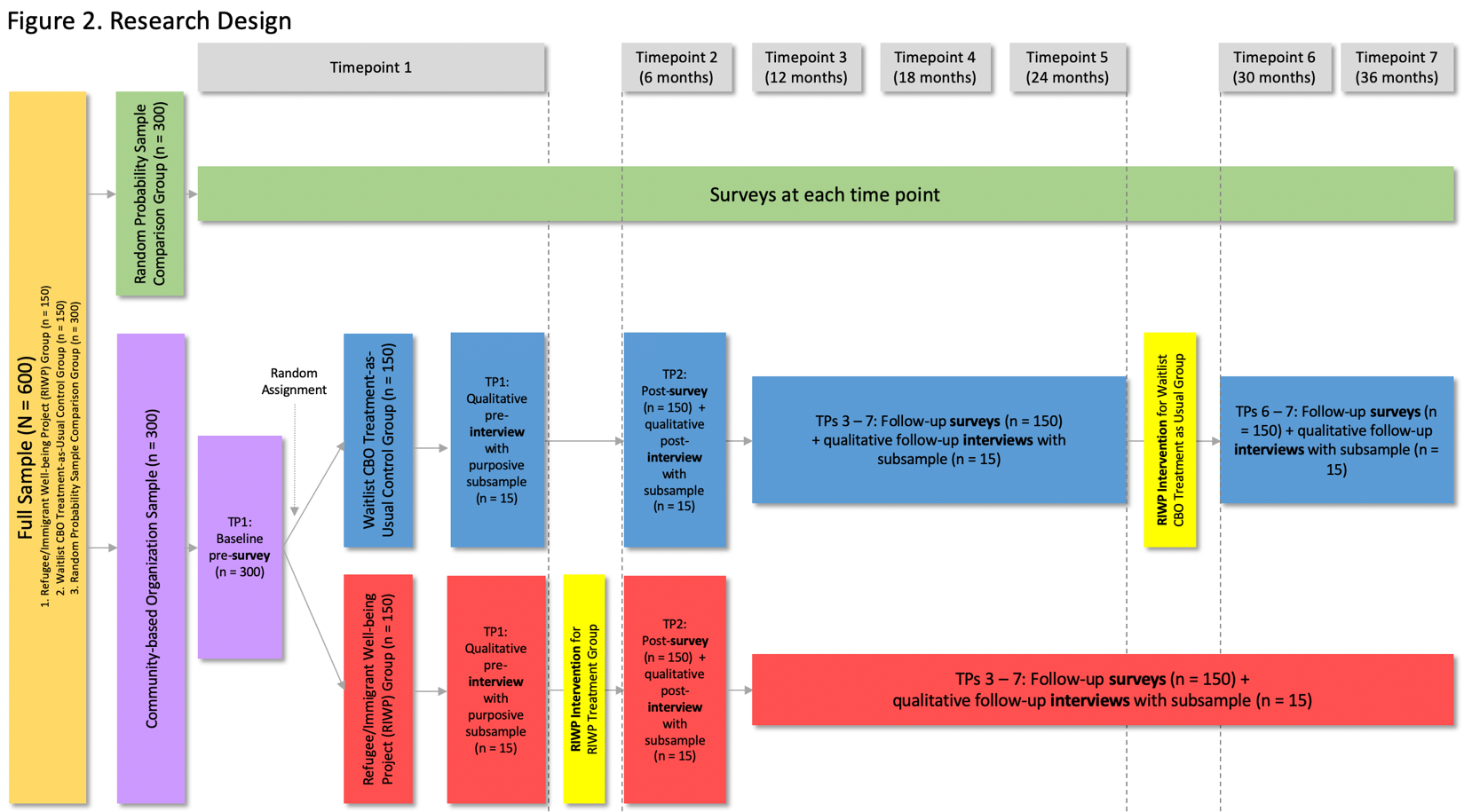


# PROJECT DESIGN

## *Target Population and Inclusion/Exclusion Criteria*

All Latinx immigrants and African refugees aged 18 and older residing in New Mexico will be eligible to participate. For the random sample of 1000 Latinx immigrants, exclusion criteria will be having used the services of one of the four community-based partner organizations serving Latinx immigrants within the past year (at time of study enrollment). For the 240 Latinx immigrants and 60 African refugees recruited through the five community-based organizations, exclusion criteria will be severe cognitive functioning problems or mental illness that is so severe as to impede participation in a group and that warrants immediate individual treatment. Exclusion will be based on review of interview data by the PI and Mr. Bill Wagner, therapist and director of Centro Sávila. Excluded adults (except those with cognitive functioning issues already being addressed or those already in treatment) will be referred immediately for individual treatment.

The proposed research will include individuals 18 years of age or older. Children ages 17 and younger will not be included in the study. The focus of the longitudinal intervention study will be on the mental health, daily stressors, and economic precarity of immigrants ages 18 and over. Their experiences of the adverse consequences of the COVID-19 pandemic are likely to be different than children’s experiences. Our focus on adults ensures that we are able to provide a developmentally appropriate intervention and utilize age-appropriate measurement.

The proposed study will be open to equal participation of men and women. Pregnant women will be eligible to participate in the study. Participant recruitment will be conducted by male and female members of the research team (including community-based organization research team members), in order to encourage participation of across all sexes and genders. All participants in the study will be Latinx immigrants or African refugees. Although the intervention involves the participation of two specific racial/ethnic groups, this focus is justified given the large disparities in COVID-19 pandemic consequences experienced by Latinx and Black populations. It is imperative to understand their experiences and the effects of multiple levels of intervention on their mental health and well-being.

## *Participant Enrollment*

Total enrollment is 1300: 1240 Latinx immigrant adults and 60 African refugee adults. The participants will be Latinx immigrant and African refugee adults (ages 18 and above) who are living in New Mexico. They will participate in the longitudinal study with 5 timepoints of survey data collection over 32 months (at 8 month intervals). Of this total number, 1000 Latinx immigrants will be a random sample comparison group recruited by LD Insights/Latino Decision. The other 240 Latinx immigrants and the 60 African refugees will be recruited through 5 CBO partners, and, in addition to the 5 same timepoints of survey data collection, will be randomly assigned after the first timepoint of data collection to the 16-week RIWP intervention or to a treatment-as-usual (TAU) waitlist control group (who will receive the RIWP intervention in Year 3). A purposive subsample of 36 participants [15 from RIWP intervention group (12 Latinx and 3 African), 15 from TAU group (12 Latinx and 3 African) and 6 from RS (random sample comparison group)] will participate in qualitative interviews at all 5 timepoints. Approximately equal numbers of men and women are anticipated. All 240 Latinx immigrant adults and 60 African refugee adults enrolled in the study through CBOs will be eligible for participation in the intervention study. Exclusion criteria will be severe cognitive functioning problems or mental illness that is so severe as to impede participation in a group and that warrants immediate individual treatment. Excluded adults (except those with cognitive functioning issues already being addressed or those already in treatment) will be referred immediately for individual treatment.

## *Recruitment and Screening Procedures*

The recruitment of 1300 participants will occur through six sites:

- Random sample of 1000 Latinx immigrant adults in New Mexico recruited by LD Insights/Latino Decisions
- 60 Latinx immigrant adults utilizing services at Centro Sávila
- 60 Latinx immigrant adults utilizing services at Encuentro
- 60 Latinx immigrant adults connected with New Mexico Dream Team
- 60 Latinx immigrant adults utilizing services at New Mexico Immigrant Law Center
- 60 African refugee adults connected with United Voices for Newcomer Rights

The random sample of 1000 Latinx immigrant adults will be recruited by LD Insights/Latino Decisions. Respondents will be randomly drawn from a mix of phone and web-based data collection, including heavy use of cell-phone numbers. Surveys will be conducted through a mixed mode format, web and phone based interviews. Surveys will be conducted in both English and Spanish at the respondent’s preference. Only respondents who self-identify as Latino or Hispanic in New Mexico would be eligible to participate. Samples are drawn randomly from the most recent publicly available household lists. Using Hispanic household list data, as identified by different commercial vendors, Spanish surname lists, and merged with third party data to secure email addresses and telephone numbers; both landline and cellphone numbers are included. One important starting point for identifying Hispanic households is to screen for Hispanic surnames using the Census Bureau list of 12,000 commonly occurring Spanish surnames. However, they do not rely on a Spanish-surname only sampling approach. Beyond the surname list, additional non-Spanish surname Hispanic households are identified by commercial market data and U.S. Census official population statistics at the census block level to more precisely identify Hispanic households in the sample. They start with as wide a net as possible and allow respondents to screen in/out if they self-identify as Hispanic or Latino. Typically, around 85% of respondents have a Spanish surname in LD Insights/Latino Decisions surveys and 15% do not, a number that tracks very closely with U.S. Census estimates. Surveying is conducted by fully bilingual interviewers. For the phone portion, respondents are greeted in both languages, and surveys are conducted in either English or Spanish, at the discretion of the respondent (see Survey Recruitment Script). Up to five callbacks are scheduled for each record. The same approach is taken for email or text message invites for online surveys. Bilingual invitations and bilingual response options are maintained for all respondents. LD Insights/Latino Decisions has a long track-record of recruiting large random samples of Latinx immigrant adults for surveys, including much larger sample sizes than the proposed 1000 participants.

The other 240 Latinx immigrant adults and the 60 African refugee adults will be recruited by CBO partners, all of whom have partnered with UNM on studies of the Refugee and Immigrant Well-being Projects and have successful histories of recruiting study participants. The staff of each CBO speak the languages of participants they will recruit (Spanish, Swahili, Kirundi, Kinyarwanda). As evident from the numbers of clients or participants engaged with each CBO per year (see Table 2), we anticipate that the recruitment goal of 60 adult participants aged 18 or older per site is very feasible. With support from the Research Coordinator Ms. Parker, Community-Based Research Specialist Mr. Ndayisenga, and graduate student Ms. Lemus, each site will share information about the study through their different activities (see Intervention Recruitment Flyer), including classes, groups, individual services, and community meetings (all of which are remote/virtual right now due to COVID-19). CBO staff will ask interested people if a study team member can call them to explain the study in more detail. During phone calls with potential participants (in their preferred language, see Intervention Recruitment Script), eligibility will be verified, the study will be explained (including random assignment to RIWP intervention or services-as-usual control group), and consent will be obtained (or reviewed for participants to consider). Participants will have the option to complete consent and the survey interview at the time of the phone call or to schedule another meeting via phone or Zoom to complete consent and the survey interview.

## *Informed Consent Process*

Potential participants will be informed (in the language of their choice – English, Spanish, Swahili, Kirundi, Kinyarwanda, or French) about the study purpose, procedures, risks, and benefits. They will be informed that their participation is voluntary, that they can decline to participate, that they are free not to answer any question, and that they can withdraw at any point without negative consequences. The alternative to participation is not to participate. Potential participants from community-based organization (CBO) partners will be assured that their decision to participate or not to participate will not affect their receipt of any services from the CBO. At the time participants consent, they will be asked to provide their name, address, phone number, email address, and the phone numbers and email addresses of at least two people who would know how to locate them in 8 months. Study participants will also be informed about the participant compensation ($25-$70 for each successive survey and $25 for each qualitative interview).

The consent form will also be read aloud to each potential participant, since some participants may not be literate. The exception to this is participants recruited through LD Insights who complete a web-based survey. They will read the consent form themselves. The consent form will be explained in detail, emphasizing the individual’s right to choose not to participate. It will be clearly stated that the individual will not be penalized in any way for choosing not to participate, and refusal to participate will in no way affect the individual’s access to services at the community-based organization that is coordinating recruitment or any services to which they are otherwise entitled. Next, each potential participant will be asked if they have any questions regarding the study. Each potential participant will be given adequate time to consider whether they want to participate in the study. The participants will then verbally indicate their consent and will be given a copy of the consent form to keep (via text or email) before the survey or interview begins. For all participants recruited by LD Insights, they will receive a text or email (along with their gift card) that has the contact information of the PI and the UNM IRB, in case they have questions or want to withdraw from the study. A **waiver of consent documentation (no signature)** is being requested because a signed consent form would be the only record linking the participant and the study, and breach of confidentiality would be the principal risk, due to the potential situation in which some participants may not have legal status in the United States. This waiver of consent documentation is also requested because interviews will most often be conducted remotely.

**HIPAA Authorization**: N/A

**Non-English Speaking Participants:** We expect that all participants in the study will complete informed consent and interviews in Spanish or Kiswahili. The consent process will be conducted by a bilingual member of the research team. The consent form and all interview guides will be translated by two study team members and back-translated by two study team members, and differences will be reconciled through in-person discussion of any discrepancies. The Spanish and Kiswahili language consent forms and interview guides will be submitted to the IRB for approval prior to study enrollment.

**Cognitively Impaired Adults/ Use of a Legally Authorized Representative (LAR):** N/A

## *Data Collection Procedures*

Quantitative survey procedure. All 1300 participants will complete 5 surveys over 32 months. LD Insights and the UNM study team will use Computer-Assisted-Telephone-Interviewing (CATI) and Computer-Assisted Self-Interviewing (CASI) protocols. CATI and CASI programming will be completed by LD Insights staff. LD Insights phone and web-based survey procedures are detailed previously. UNM bilingual study team members will follow a similar protocol (CATI over the phone or web-based CASI emailed to participant), depending on participant preference and literacy. Compensation for participants’ time for each survey will start at $25 and increase as follows: T1 $25, T2 $40, T3 $50, T4 $60, and T5 $70. Increasing payments over time improves retention in longitudinal studies.^82,83^ The survey will include well-validated measures from PhenX Toolkit, except when noted due to lack of appropriate measure (see Table 1). Our research team has extensive experience ensuring culturally appropriate measurement of mental health; we expect the measures we have chosen to be relevant and efficient and to maximize our opportunity to understand intervention effects on multiple dimensions of psychological distress. Single-item questions from PhenX to measure demographic, control, and moderator variables are: biological sex assigned at birth, gender identity, sexual orientation, age, race/ethnicity, birthplace, years living in U.S., annual family income, marital status, employment status, educational attainment, health insurance coverage, household roster/relationships, and English proficiency. Survey questions will also obtain additional information needed for propensity score matching by asking about factors likely to predict involvement with CBO partners: length of residence in NM, having children in schools, use of community services, and religious service attendance. Most measures have been used with Spanish-speaking immigrants; we will translate measures into Kiswahili using a team approach, following the TRAPD (Translation, Review, Adjudication, Pretesting, and Documentation) process.^70,71^ The survey will be piloted in all languages to ensure comprehension, appropriateness, and length of 30-40 minutes or less.

| Table 1. Quantitative Measures | | | |
| --- | --- | --- | --- |
| **Variable** | **Measure** | **Detail** | # of Items & Psychometrics |
| Psychological Distress | 1. DSM Level 1 Cross-Cutting Symptom Measure Adult^72^  2. COVID-19 & MH Impacts^73^ | - Broad Psychopathology measure; 13 psychiatric domains  - Emotional health, worries | - 23 items; 2-week timeframe; reliable & valid; clinically useful, PhenX  -13 items; PhenX |
| Daily Stressors | 1. Perceived Stress Scale^74^  2. Effects of COVID-19 Outbreak^75^ | - Current stressors  - Stressful effects & behaviors | - 10 items; reliable & valid; PhenX  - 11 items; PhenX |
| Economic Precarity | 1. Job Insecurity - General Social Survey 2018^75^  2. RAND American Life Panel Impacts of COVID-19 Survey | - Measures likelihood of losing job and obtaining another job  - COVID-19 Related Household Finances | - 2 items; PhenX  - 6 items; PhenX |
| Access to Resources | Satisfaction with Resources Scale^76^ | - Assesses access to resources in 12 domains | - 12-items; used with newcomers |
| Social Support | Medical Outcomes Study Social Support Survey, 8-item version^77^ | - 2 subscales: instrumental and emotional | - 8 items; reliable & valid; PhenX |
| Cultural Connectedness | Language, Identity and Behavior Acculturation Scale,^78^ short | - Multiple dimensions of cultural connections | - 16 items (parallel versions of 8 items for U.S. culture and home culture) |
| Mental Health and Health Services Use | 1. Composite International Diagnostic Interview (selected questions)^79^  2. Access to Health Services^80^ | - Measures counseling and medications received from a helping professional  - selected items | - 2 items; reliable, valid & used with immigrants  - 2 items on health services use; PhenX |
| Discrimination | Experience of Discrimination (EOD) Scale^81^ | - Measures discrimination in 9 domains | - 9 items; reliable & valid; used with Latinx & Black adults; available in Spanish |

Qualitative interview procedure. Dr. Hess will lead a team of bilingual interviewers who will conduct semi-structured qualitative interviews with a subsample of 36 participants recruited by CBOs [15 from RIWP intervention group (12 Latinx and 3 African), 15 from TAU group (12 Latinx and 3 African) and 6 from RS (random sample comparison group)] at all 5 timepoints. Interviewees will be selected based on a combination of searching for maximum variation and specific criteria,^84^ including race/ethnicity, biological sex, sexual orientation, SES and CBO connection, after completion of timepoint 1 surveys and random assignment to RIWP or TAU group. Numerous qualitative studies have found that theoretical saturation is reached with sample sizes of 12-26 for each population segment.^85,86^ Except for the first timepoint, qualitative interviews will coincide with the surveys and will occur after completion of the survey. Interviews will be 45-60 minutes, and will be conducted via Zoom or phone until it is safe to do otherwise, at which time participants will be offered the option of completing them in-person or via Zoom or phone. Participants will receive $25 for each interview. With participants’ consent, the interviews will be digitally recorded. Dr. Hess will also conduct semi-structured group interviews with CBO representatives to learn about contexts and challenges of RIWP implementation. In addition, newcomers in RIWP and their undergrad partners will participate together in a post-intervention qualitative interview to explore their experiences (e.g., what partners learned from and taught each other, best and most difficult things about working together). These interviews serve a different purpose from the other qualitative interviews – to enable pairs to have mutual dialogue about their experiences. Topics will complement survey questions and include mental and physical health, current stressors, cultural identity, discrimination, and health and CBO services that participants find helpful. RIWP participants will also be asked about their experience in the intervention and changes over time in their lives, health and mental health, stressors, families, and communities that they relate to intervention participation.

Random assignment. Randomization of the CBO-recruited sample (n=300) into RIWP or treatment-as-usual waitlist control group (TAU) will occur after completion of timepoint 1 surveys and will be based upon a computerized algorithm that stratifies randomization by CBO site.

RIWP implementation. RIWP will be implemented with 30 participants from each CBO site (n=150) from Months 5-12. RIWP will be integrated into existing activities and services in different ways at each site, with the goal of creating a sustainable intervention and multilevel change. All RWIP activities (Learning Circles and advocacy) will be conducted remotely for now. Once state and UNM COVID restrictions are lifted, some in-person Learning Circles and advocacy meetings may occur. The treatment-as-usual waitlist control group will participate in RIWP after two years (3 timepoints) of data collection, from Months 29-35. RIWP involves a sustainable and replicable partnership model between refugees, CBOs, and universities (see Figure 1 for conceptual model). Newcomer families and undergraduate advocates work together for 16 weeks to: a) increase newcomers’ abilities to navigate their new communities; b) improve newcomers' access to community resources; c) enhance meaningful social roles by valuing newcomers' cultures, experiences, and knowledge; d) reduce newcomers' social isolation; and e) increase community responsiveness to newcomers. RIWP is implemented by university students enrolled in a 2-semester course, and has two elements: 1) Learning Circles, which involve cultural exchange and one-on-one learning opportunities; and 2) Advocacy, which involve collaborative efforts to mobilize community resources related to health, housing, employment, education, and legal issues and to create policy/system changes. Meeting in weekly Learning Circles, newcomers and their student partners learn from each other during cultural exchange time designed to facilitate sharing of cultural and intergenerational knowledge and to help newcomers recognize their contributions and potential to effect changes in their communities. During one-on-one learning time, newcomer and student participants practice English, fill out job applications, and engage in other activities newcomers want to pursue to expand knowledge, improve skills, or accomplish goals. RIWP is holistic, multilevel, and strengths-based, and has an explicit social justice orientation that is informed by an ecological perspective. A holistic intervention is essential because it addresses the multiple sources of newcomers’ psychological distress, including psychological (past traumas), material (poverty, lack of access to resources), physical (physical ailments from war, violence and deprivation), social (loss of meaningful social roles and social support), educational (limited English proficiency, literacy), and cultural disconnection from traditional cultural practices). A strengths-based perspective is important because newcomers have survived in the face of tremendous hardships and have numerous strengths on which to build, including cultural knowledge (and often extensive cross-cultural experience), resourcefulness (experience operating in resource scarce situations), effective coping strategies (that have enabled them to survive), multilingual abilities (many arrive already speaking two or more languages), and often strong family and community bonds and support. Newcomers are often pathologized by well-intentioned service providers and systems, rather than recognizing that most are resilient people who have faced abnormally difficult circumstances. Not only is it crucial for newcomers’ mental health and well-being to acknowledge their expertise and build upon their strengths, but also their knowledge and experiences are assets from which other Americans can benefit. Thus, mutual learning is an intentional and core component of RIWP. This emphasis also ensures that RIWP is appropriate for multiple linguistic and cultural groups who can participate simultaneously and learn from one another as well as from longer-term residents of the U.S. Guided by ecological theory, RIWP focuses not only on newcomers adjusting to their new contexts, but also on changing environments when they are inequitable or constraining (e.g., mobilization or redistribution of resources). Ensuring that RIWP creates change at multiple levels is important because newcomers have urgent learning, resource, and health needs that must be addressed at the individual level in order to enable them to work collectively with others to create community and structural changes that address the root causes of mental health disparities and social inequities, which result in sustainable social change.

Community-Based Participatory Research (CBPR) Partnership. This research is built on a longstanding partnership with five community-based organizations (CBOs) that engage in mental health, legal, educational, and community organizing efforts with Latinx immigrants and African refugees: Centro Sávila (<http://www.centrosavila.org>), Encuentro (<http://www.encuentronm.org/>), New Mexico Dream Team (<https://www.nmdreamteam.org>), New Mexico Immigrant Law Center (<http://nmilc.org>), and United Voices for Newcomer Rights/Refugee Well-being Project (<https://rwp.unm.edu>). This diversity of organizations will enable us to reach Latinx immigrants and African refugees who are facing stressors and high psychological distress due to the COVID-19 pandemic. These partner CBOs are already involved in our collaborative study (core research project of U54MD004811) to adapt and integrate RIWP into their work with Latinx immigrants (see LOS). As in our current practice, one staff from each organization will be a member of the research team, which meets twice per month and make decisions on all aspects of the study, including: finalizing survey questions and interview guides, adapting/integrating RIWP into their sites, refining data collection methods, participant recruitment, RIWP implementation, data analysis, and dissemination. These staff are paid through subawards and contracts to each organization. In the past 3 years, we have been a highly productive team. We have developed an innovative bilingual, participatory data analysis method together,^60^ and disseminated our work through conference presentations, manuscripts, and research and policy briefs. In addition, CBO directors and organization members will participate in a community advisory council (CAC) that provides broader input and guidance on a less frequent basis (3 meetings per year). There will be 10 community CAC members in addition to CBO directors and staff, an appropriate size for diversity in terms of age, gender, and other aspects of social identity and experience, while ensuring that the CAC is feasible and maximizes participation of each member. CAC members will receive an honorarium of $50 per meeting. CBO partners have subawards or contracts, each with a budget of $30,000 per year for the 5 years of the study. This dedicates $150,000 to each CBO (total=$600,000) for their time and involvement.

## *Anticipated End Date*

Data collection will be completed by December 31, 2024, and de-identified by June 30, 2025.

## *Project Location(s)*

This research is built on a longstanding partnership with five community-based organizations (CBOs) that engage in mental health, legal, educational, and community organizing efforts with Latinx immigrants and African refugees: Centro Sávila (<http://www.centrosavila.org>), Encuentro (<http://www.encuentronm.org/>), New Mexico Dream Team (<https://www.nmdreamteam.org>), New Mexico Immigrant Law Center (<http://nmilc.org>), and United Voices for Newcomer Rights/Refugee Well-being Project (<https://rwp.unm.edu>). This diversity of organizations will enable us to reach Latinx immigrants and African refugees who are facing stressors and high psychological distress due to the COVID-19 pandemic (see attached Letters of Support).

## *Participant Compensation*

All 1300 participants will receive compensation for each of the five 30-40 minute surveys they complete, starting at $25 and increasing as follows: T1 $25, T2 $40, T3 $50, T4 $60, and T5 $70. The subsample of 36 participants who participate in qualitative interviews will receive $25 for each of the 5 timepoints of qualitative interviews. These will be paid in the form of gift cards, which will be mailed, emailed, or texted to participants (depending on participant preference).

## *Project Resources*

The facilities and other resources available to the PI and the research team at the primary performance site include the necessary support, equipment and materials to undertake and complete the proposed research project successfully. The PI Dr. Goodkind’s office and lab are located in the UNM Department of Sociology and UNM Center for Social Policy. Dr. Hess has her office and research space in the UNM Health Sciences Center Department of Pediatrics Division of Prevention and Population Sciences (DPPS). Dr. Sanchez’s office and research space are in the UNM Center for Social Policy, as well as Mr. Ndayisenga and Ms. Parker’s offices. Drs. Van Horn and Lardier’s offices and research spaces are in the Department of Individual, Family, and Community Education (IFCE). The Departments of Sociology and Political Science, Center for Social Policy, DPPS, and IFCE all have shared equipment for staff to use such as copiers, printers, Zoom telecommunication software and equipment, fax machines, scanners, audio-visual equipment, and speaker phones. Computer resources, including desktop PCs and printers, are available for most of the proposed staff on this project – some funding is included in the proposal for desktop and laptop computers. Software for routine project tasks, such as word processing, email, and internet access is available, as well as most data management and statistical software. In addition to shared equipment, there are administrative support staff and computer technical support to assist investigators in their daily work. Physical space for project material storage and meetings are also available in the PIs lab space and in the Center for Social Policy. **Computing:** One primary resource required for this study is the computers and software needed for complex data analyses and Monte Carlo simulations. The Methodology Group @ UNM has dedicated space in a server room which is adjacent to their physical space allowing 24 hour a day access. The server room has a security alarm and camera system, cooling, direct connection to the university backbone, and 220 amp power. The group currently has a 24U rack in the server room with UPS, KVM switch, and one 24-core server with 256 GB of RAM, one 44 core server with 256 GB of RAM, and a fileserver to meet the needs of this grant and other quantitative research in the group.

# EXPECTED RISKS/BENEFITS

## *Potential Risks*

There are no physical risks that can be reasonably anticipated. Completing the interviews and intervention is non-invasive and voluntary.

Psychological risks are minimal because participants will answer questions about their current mental health and current stressors. These questions could potentially be upsetting. Participants can skip any questions they do not want to answer and can end their participation at any time.

Social risks are minimal because participants in the intervention will share some of their experiences in a group setting. However, they make the voluntary decision to do so and do not have to share anything that they do not feel comfortable sharing. All interviews will be conducted in private settings and the information collected will be kept confidential, to the extent allowable by law.

There are no financial risks that can be reasonably anticipated. The study does not involve any financial cost to participants. The intervention (Learning Circles and advocacy) are flexible and designed not to interfere with participants’ work schedules; transportation and child care are provided when not meeting remotely.

Participation in this research study will involve a loss of privacy as participants are being asked to share personal information. However, every effort will be made to maintain confidentiality and privacy of all participants’ responses. We will ask participants to keep personal information that is shared in the Learning Circles confidential. One of the explicit goals of the intervention is to develop trust and strengthen relationships among group members.

The primary protection against potential risks is a data security plan that includes physical, administrative and technical aspects for confidentiality and privacy of the data, including personal identifiers, which will be approved by the University of New Mexico IRB prior to the study. Of particular importance will be the appropriate training of all study staff, including investigators, research coordinator, RAs, and interviewers in maintaining confidentiality and privacy of all data, in addition to their formal training from Collaborative Institutional Training Initiative (CITI), which is required by UNM IRB. All information will be kept confidential to the extent allowable by law. No names or identifying information will be included on interviews, audio digital recordings, or transcripts. We will not inquire about immigration status at any point during the study.

Each participant will be assigned a unique numerical identifier (five-digit ID number) and this code will be placed on the interview forms, audio digital recordings, and transcripts. The master list linking participants to ID numbers will be kept separate from the data set in a locked file cabinet in Goodkind’s (PI) lab and on her password protected computer. No identifying information will be included in any reports or presentations resulting from this study. Furthermore, only general themes appearing across participants will be reported. Intervention participants will also be asked to keep all information shared in the Learning Circles confidential. All interviews and intervention activities will occur remotely or in the community (at participants’ homes, community centers, community organizations, or other convenient location) in order to ensure that research activities occur in convenient, comfortable settings and to minimize any potential risks or discomfort. Because the RIWP intervention will be open to all interested immigrants and refugees, participants who participate will not be identified as having particular mental health problems.

It is possible that study participants may be hesitant to participate in our study because of their immigration legal status or fear that we may disclose their identity to government/federal agencies. Our team has substantial experience in recruiting and consenting Latinx populations of mixed immigration status, including undocumented immigrants. Therefore, we will ensure that we have a Certificate of Confidentiality (CoC) from NIH. As it states on the NIH website “Certificates of Confidentiality are issued by the National Institutes of Health (NIH) to protect identifiable research information from forced disclosure. They allow the investigator and others who have access to research records to refuse to disclose identifying information on research participants in any civil, criminal, administrative, legislative, or other proceeding, whether at the federal, state, or local level. By protecting researchers and institutions from being compelled to disclose information that would identify research subjects, Certificates of Confidentiality help achieve the research objectives and promote participation in studies by helping assure confidentiality and privacy to participants.” We will explain this Certificate of Confidentiality and its protections and possible limitations to participants as part of our informed consent process to assure confidentiality and privacy of their data. Our research study team has extensive experience conducting studies with Latinx populations of mixed immigration status and with utilizing protocols that we have found effective for minimizing risks while working with these populations. These include: 1) not asking about immigration status; 2) obtaining a Certificate of Confidentiality from NIH; 3) obtained oral informed consent; and 4) minimizing collection of identifiable and private data as much as possible.

## *Benefits*

The risks of this project are reasonable when compared to the expected benefits to the participants and others. The potential benefits of this study are great. Immigrant and refugee adults may have the opportunity to learn, work together to address community issues, increase their social support, and improve their access to community resources. Based on the previous studies of the intervention model, it is expected that the intervention will reduce distress and increase protective factors among participants. Latinx immigrants and African refugees in New Mexico and throughout the United States continue to struggle and experience high rates of psychological distress. Thus, this intervention has significant potential for improving the lives of these groups. Furthermore, if the intervention demonstrates feasibility, acceptability, appropriateness, and effectiveness, it can be disseminated throughout the United States to improve the lives of other immigrants and marginalized populations.

The knowledge that will be acquired from this study will further our understanding of community-based mental health interventions for marginalized populations that address social-structural determinants of mental health and will contribute to our ability to reduce the high rates and disproportionate burden of COVID-19 adverse consequences experienced by socioeconomically disadvantaged Latinx immigrants and African refugees in the United States. As stated previously, this knowledge has important implications for Latinx immigrants and African refugees, as well as all other immigrants and refugees who resettle in the United States and broader Latinx and Black populations. In addition, the knowledge generated from this study is important in furthering the fields of research on immigrant and refugee mental health, health disparities, and structural inequities. We will be studying the effects of involvement with community-based organizations and structural interventions (policies to support immigrants and refugees during the pandemic) in addition to the RIWP intervention. It is particularly important to design, implement, and evaluate innovative, multilevel mental health interventions for immigrants and refugees that address the social and structural determinants of health because limited research exists in this area. There are also specific benefits to communities and broader society in terms of the data that are collected, because the data can further our understanding of the COVID-19 pandemic and post-pandemic experiences of Latinx immigrants and African refugees and can demonstrate that universities in partnership with communities can provide evidence-based interventions. Thus, the potential benefits to participants and other immigrants and refugees and the knowledge gained outweigh the minimal risks to participants.

## *Privacy of Participants*

All consent procedures and interviews will be conducted via private Zoom or in private settings with only the interviewer and participant present (unless the participant would like to include other people during the consent process). We will ensure that these private rooms have doors that can be closed to further protect participants’ privacy. Participants can choose to complete the interviews in the setting they feel most comfortable (e.g., via Zoom, in their home, the community organization, or other community setting). To further protect participants’ privacy, the quantitative components of the interviews are completed using Computer-Assisted-Telephone-Interviewing (CATI) and Computer-Assisted Self-Interviewing (CASI) protocols. Learning Circles will be held in private Zooms or private rooms with doors at the community organizations or community centers. Learning Circle participants will be asked not to share any information from the group outside of the Learning Circles.

## *Unanticipated Problems/Adverse Events*

Data and safety monitoring will be overseen by the PI and co-investigators Drs. Vasquez Guzman, Hess, Van Horn, Lardier, Sanchez and Wagner (licensed mental health therapist and Executive Director of community partner CBO Centro Sávila), and the UNM IRB. Learning Circle facilitators and interviewers will be required to report any adverse events or unanticipated problems to the PI within 24 hours, who will discuss them with Mr. Wagner, document them in writing, and report them to the UNM IRB immediately (within 3 calendar days). The PI, the co-Is, and Mr. Wagner will meet monthly to review the research protocol, data on recruitment and retention, quality control, adherence, and adverse events and safety, as well as data management systems and study results. They will monitor the well-being of participants as reported in the periodic interviews and through any concerns raised by interviewers during weekly research team meetings. Monitoring of participants’ well-being by intervention staff (research coordinator and RAs) will occur on an on-going basis as well, and they will also be asked to share any issues or concerns at weekly research team meetings.

## *Participant Complaints*

Participants will have multiple channels for communicating potential complaints and/or requesting additional information. In addition to the phone number for the PI and the UNM IRB, which will be provided on the consent form, participants will be able to discuss complaints and/or submit them anonymously in writing to the interviewers and intervention facilitators. Furthermore, participants can also discuss any concerns with staff at the community partner organization through which they are participating. These staff will have a form for documenting concerns/complaints and submitting them confidentially to the PI.

# PROJECT DATA

## *Data Management Procedures and Confidentiality*

All qualitative interviews will be digitally recorded (if participant agrees) and transcribed verbatim. The audio digital recordings will be kept on a password-protected UNM Shared Drive (which the PI already has in her lab). The only people with access to file on the Shared Drive with the audio digital recordings will be the PI and other members of the qualitative research team (Drs. Hess and Guzman Vasquez and Ras). The audio digital recordings will be destroyed (by deleting the files) once the study is completed. The transcripts will be electronic only and will not include any identifying information. If participants use any names during the qualitative interviews, these will be changed to pseudonyms in the transcripts.

The quantitative data will be collected via Computer-Assisted-Telephone-Interviewing (CATI) and Computer-Assisted Self-Interviewing (CASI), which means it will be entered directly into password-protected laptops. Thus, there will be no paper records of quantitative data. The CATI and CASI data will include ID numbers only and will be transferred from the laptops to the Secure Shared Drive as soon as it is collected.

The only individually identifiable private information that will be collected from participants is name, address, and phone number for tracking purposes. The PI, co-investigators, research assistants, and interviewers will have access to participants’ names, addresses, and phone numbers. However, this information will be kept separate from all interview and intervention data collected. Each participant will be assigned a unique numerical identifier (five-digit ID number) and this code will be placed on the interview forms, audio digital recordings, and transcripts. The master list linking participants to ID numbers will be kept separate from the data set in a locked file cabinet in the PI’s lab and on the PI’s password protected computer. This master list will be destroyed prior to study closure.

All information will be kept confidential to the extent allowable by law. No names or identifying information will be included on interviews, audio digital recordings, or transcripts. We will not inquire about immigration status at any point during the study. No identifying information will be included in any reports or presentations resulting from this study. Furthermore, only general themes appearing across participants will be reported. Intervention participants will also be asked to keep all information shared in the Learning Circles confidential.

As stated on the NIH website (<https://humansubjects.nih.gov/coc/NIH-funded>), “Per Section 2012 of the [21^st^ Century Cures Act](https://www.gpo.gov/fdsys/pkg/PLAW-114publ255/pdf/PLAW-114publ255.pdf) as implemented in the [2017 NIH Certificates of Confidentiality Policy](https://grants.nih.gov/grants/guide/notice-files/NOT-OD-17-109.html), all ongoing or new research funded by NIH as of December 13, 2016 that is collecting or using identifiable, sensitive information is automatically issued a CoC (Certificate of Confidentiality).

## *Data Analysis/**Statistical Considerations*

Quantitative data analysis. This longitudinal study design will use random effects (multilevel) models, which account for both repeated measures of individuals and the fact that in one arm individuals are clustered within CBOs. These models allow for outcomes to be missing under the MAR assumption (data is missing at random conditional on the observed outcomes and covariates),^89–91^ which we expect will be adequate. If there is a substantial amount of missing data for covariates or evidence that a more inclusive imputation model should be used, then we will use multilevel multiple imputation implemented via MITML package.^92^ Model assumptions will be evaluated using the comparison group for each analysis, and any modification to the analyses to address violations will be made before final analyses using only comparison group data.

This study is specifically designed to be sensitive to the changing impacts of the pandemic and the effectiveness of the multilevel interventions in reducing those. Thus, we propose 5 data collection points, every 8 months over 32 months. Our primary outcomes – psychological distress, daily stressors, and economic precarity – tend to be moderately stable^93–98^ but may also be impacted by shocks such as losing a job or getting COVID-19. Given the unknown trajectory of the pandemic and the path of economic recovery, we do not know a priori what the best approach to use for the longitudinal data will be. Therefore, our proposal is to use those in the random comparison sample in order to explore longitudinal models which will capture a large portion of the variability in outcomes over time while also maximizing power to find treatment effects. For example, modeling effects on mean levels of the outcomes post baseline would involve only 1 model parameter but would miss much of the nuance in the data. The comparison respondents will allow us to examine possible alternatives (linear change, piecewise change, adjustments for time-varying events including unemployment and COVID infections) in data without treatment effects. We will conduct analyses and publish a paper which examines fit of alternative longitudinal models to the data, and we will conduct additional power analyses before choosing the approach which best balances model fit and power. For the remainder of the data analysis section, we assume a relatively simple model with an intercept and linear change, while power analyses assume a simple pre-post design which will result in conservative estimates of power.

**Aim 1.** Test the effects of the RIWP intervention to reduce adverse consequences of the COVID-19 pandemic. The principal objective of this study is to test the efficacy of the RIWP intervention for participants recruited from CBOs. Because only individuals recruited from the CBO sample are eligible to be randomized to receive the RIWP intervention or treatment as usual, analysis for this aim includes only CBO participants. To examine the effects of the RIWP intervention, we propose a linear model with random effects for individuals. As randomization happens within CBO, there is not an explicit random effect for this included, but we do propose to use sandwich estimators,^99,100^ to adjust standard errors for any unexpected impacts of this clustering. In mixed notation the model for psychological distress is:

${Distress}_{ti}= B_{00}+{B_{01}\left( RIWP \right){+B}_{02}\left( Time \right){+B}_{03}\left( Time*RIWP \right){+\boldsymbol{B}}_{\mathbf{0}\boldsymbol{N}}\left( \boldsymbol{covariates} \right)+ r_{0ij}+r_{2ij}(Time)+ e}_{ij}$

where participation in the RIWP plus CBO is indicated by a dummy code for RIWP. Because baseline data is collected before randomization, time is centered at baseline and our aim is answered by B_03_ which is the difference in slopes of levels of distress, adjusted for covariates, between those who experience RWIP+CBO intervention and those in the CBO group.

**Aim 2**. Test the effects of engagement with CBOs to reduce adverse consequences of the COVID-19 pandemic. Analyses will use both the CBO sample and the random sample of Latinx respondents from across the state. The primary predictor is whether the participant is recruited through the CBO or random community sample. Our analyses test whether respondents recruited from CBOs differ from those in the random sample in outcomes and whether those differences change over time. While the comparison sample is a random probability sample of Latinx immigrants, the CBO sample consists of respondents already connected to a CBO, thus we propose to use propensity score matching^101,102^ with data collected at baseline to match those in each sample. The matched samples used for data analysis will adjust for systematic differences between the random sample and those who engage with CBOs. Our baseline measures have been chosen to include variables which are likely to predict an individual’s exposure to a CBO. To examine the effects of engagement with CBOs, we propose a longitudinal model with random effects for both individuals and CBOs. The model will also adjust for effects of the RIWP intervention observed in Aim 1. We note that the adjustment for clustering within CBOs only takes place for those in the CBO arm; in the comparison arm where there is no nesting, these random effects are assumed to be zero.^103,104^ In mixed notation the model for psychological distress is:

${Distress}_{tij}= B_{000}+{\gamma_{001}\left( CBO \right)+{B_{100}\left( Time \right)+\gamma_{101}\left( CBO*Time \right)\boldsymbol{+B}}_{\boldsymbol{010}}\left( \boldsymbol{covariates} \right)+{B_{020}\left( RIWP \right)+B_{030}\left( RIWP*Time \right)+ u_{00j}+u}_{10j}\left( Time \right)+r_{0ij}+r_{1ij}(Time)+ e}_{tij}$

where CBO is an indicator indicating whether individual *i* is engaged with the CBO, Time is a variable indicating the time of observation in years. For this analysis, Time will be centered around the midpoint of the study because engagement with the CBO is ongoing from before the study began. Thus, our aim is answered by Y_001_ which is the difference in mean levels of distress, adjusted for covariates, between those who experience the CBO and those in the random sample and by Y_101_ which is the difference in slopes over time between those who experience the CBO and those in the random sample.

**Aim 3.** Test the effects of RIWP and engagement with CBOs to increase access to benefits of structural interventions (local/state relief-related policies). For this aim, the outcome will be a binary indicator for whether each participant accessed the structural interventions which are implemented. The analyses will be similar to Aim 2, but will use a logit link and consequently drop the e_tij_ term. As mentioned above, while this is written to assume linear changes, we have proposed a process for selecting the longitudinal model to be used with data from the comparison sample and using statistical simulations to evaluate power of the different alternatives.

**Secondary Aims.** (a)   Examine mechanisms of intervention effectiveness. This secondary aim will be examined through structural equation modeling (SEM) to provide tests of mediation effects through which outcomes are impacted by hypothesized mechanisms. The same models for changes in outcomes which are developed on the random sample^110^ will be used in these analyses. These analyses assess whether differences in outcomes between treatment groups are due to changes observed in protective factors from baseline to post-intervention.^111^ Significant indirect effects are anticipated linking the RIWP intervention with lower follow-up levels of distress, stressors, and economic precarity, via pre-to post-intervention increases in protective factors. Bootstrapped standard errors^105^ will be used to derive confidence intervals for these mediated effects; standard errors will be adjusted for clustering of participants within CBOs using sandwich estimators.^106^ (b) Explore whether race/ ethnicity, biological sex, gender identity, sexual orientation, age, and SES moderate intervention impact. For this secondary aim, stratification variables will be incorporated as level 2 covariates in the models described above. Moderation will be assessed via interactions between the multiple levels of treatment and each of these moderators.^107^ (c) Track local/state policy changes to obtain preliminary estimates of effects of these structural interventions on mental health, stressors, and economic precarity using propensity score matching. This aim will evaluate the impact of receiving the structural interventions which are the outcome of Aim 3 on mental health, stressors, and economic precarity. We propose to use propensity scores, but in this case where the treatment is receiving a structural intervention. Propensity scores will be developed using baseline data to predict the probability of receiving a structural intervention. We will then assess the impact of these interventions using matched samples in random effects models. Mediation will be examined using Sobel’s test to examine whether RIWP effectiveness had an indirect effect on mental health, stressors, and economic precarity through structural interventions (local/state policy changes).^108–110^ This is a secondary aim because, while important, there is not enough information to assess power a priori.

**Power.** Power estimates were obtained using a simplified pre/post design which results in conservative estimates because it does not consider the multiple assessments. Based on preliminary analyses from similar samples^42^ we assume that the correlation over-time between the outcome at baseline and posttest will be .68. We also assume that attrition in the CBO sample will be 10% based on prior RIWP research,^40^ but we allow for attrition of 30% in the random sample of Latinx immigrants, which is an appropriate attrition expectation that has limited bias in means.^111^ Finally, based on the previous trial of RIWP, observed effect sizes for significant effects were small to moderate (ranging from Cohen’s D of .30 to .45),^42^ thus our objective was to determine the power for an effect size of .3 or more. For **Aim 1,** simulations adjusting for baseline values on the outcome estimate **power to be .91** to detect an effect size of .3. For **Aims 2 and 3**, there is not an established analytic approach to power and so we used statistical simulations (conducted in R, with analyses run using LME for the continuous outcome and GLMER for the binary outcome). These analyses assumed random effects for CBO and ICCs of .03 based on previous literature examining outcomes clustered within neighborhoods.^112–114^ For the **Aim 2** simulations, data were generated according to an ANCOVA model and appropriate type II error control was examined by running simulations with an effect size of 0. Across 1000 simulations, we estimate that power to detect an effect size of .30 is **.92**. For **Aim 3**, power was estimated for a binary outcome with probability of an individual in the comparison group receiving state level interventions of .2, and the level 2 variance was .10, which is approximately equal to an ICC of .03. We estimated power to detect an odds ratio of 2 for the effect of CBOs on receiving state level of interventions as **.82**. Last, power estimates were obtained for secondary aims using a simple ANCOVA formula in G*Power.^115,116^ For **secondary aim a,** power was **.90** for an effect size of .3, with power estimates ranging from .70 (d = .28) to .95 (d = .36). For **secondary aim b**, power was **.87**, with power estimates ranging from .70 (d = .24) to .95 (d =.33). For **secondary aim c,** estimates displayed power of **.80**, with effect size of .23, with power estimates ranging from .70 (d = .19) to .90 (d = .28). Thus, the study has strong power to detect the hypothesized effects in Aims 1 and 2, and moderate power to detect effects for Aim 3. Secondary aims also have moderate to strong power to detect effects.

Qualitative data analysis. Qualitative data will contribute to addressing all aims, including effects of RIWP and CBO involvement on primary outcomes of psychological distress, daily stressors and economic precarity and secondary protective factor outcomes; understanding how RIWP and CBOs may increase newcomers’ access to benefits of structural interventions; direct effects of structural interventions; how RIWP newcomer and student participants contribute to structural changes through efforts to enact relief policies; and the different contexts and processes of RIWP implementation. The analysis of qualitative data is not discrete from the process of collecting it. Thus, we will take detailed notes on process and content after each interview. We will record insights, questions, and reflections, and consider the implications of what we are learning. Once interviews are completed, the Spanish audio will be professionally transcribed and checked for accuracy. Using a participatory data analysis process developed by the RIWP partnership, we will use retreats for CBO and community members to interpret text and identify important themes. Next, we will open-code the data using preliminary codes identified by the large group. The UNM team will also identify other emerging themes, and all themes will be consolidated into a codebook. The code structure will then be applied by at least 2 independent coders to the transcripts. Further consolidation of the coding structure will occur through focused coding^117^ of prominent themes and determination of which themes are common to most participants and which suggest discrepancies. We will also use focused coding to begin to determine how themes are related to one another, and to create visual models of these relationships.^118^ Analysis, organization, and management of the qualitative data will be facilitated by NVivo software, which allows for rapid coding and development of a coherent coding taxonomy. Drs. Hess and Vasquez Guzman will oversee the participatory data analysis process.

Overall, the qualitative study components will serve a complementary and explanatory role as an associated embedded strand in conjunction with the primary quantitative design. We will use a constructivist grounded theory approach^117^ to explicate processes, in this case change related to the multiple levels of intervention. Theory is “grounded” in the data themselves. Constructivist grounded theory therefore uses a combined inductive and deductive approach, coupled with the recognition that study participants and researchers ‘co-construct’ data, thus making it a good fit for CBPR studies.^117^ A key aspect of this approach is its iterative nature; therefore, analyses will be ongoing and inform questions asked in subsequent interviews.

Integration of mixed methods data. In a mixed methods study, the integration of quantitative and qualitative data is paramount.^119^ Our study has a complex mixed method design that includes a series of connected projects as reflected in Aims 1-3.^120^ In this *mixed method intervention design*, the quantitative design is primary and the embedded qualitative strand is a secondary source of data at all timepoints. The qualitative function is to provide convergence, complementarity, and expansion on quantitative data.^121^ Convergence occurs when qualitative and quantitative methods are used to answer the same research questions (e.g., how outcomes change over time). Qualitative data will also be used to expand on quantitative findings. Quantitative and qualitative data will be integrated in two primary ways.^119^ First, the data will be *connected*wherein quantitative data (e.g., demographic information; psychological distress) will be incorporated into our qualitative dataset to inform qualitative data collection from participants. Second, data will be *merged* to explore individual and structural policy change. The PI and co-I’s will have monthly meetings throughout data collection and analysis to ensure that processes are integrated, and findings are informing the complementary approaches.

## *Participant Withdrawal*

Participants are free to withdraw at any point from the study. If a participant indicates their desire to withdraw from the study, we will not collect any further interview data from the participant. Any data collected previously from a participant who withdraws from the study will be destroyed [removed from the quantitative and qualitative databases and not included in analyses.

# PRIOR APPROVALS/REVIEWED AT OTHER IRBS

N/A

# REFERENCES

1. Budiman A. *Key Findings about U.S. Immigrants*.; 2020. Accessed November 21, 2020. https://www.pewresearch.org/fact-tank/2020/08/20/key-findings-about-u-s-immigrants/

2. Holtgrave DR, Barranco MA, Tesoriero JM, Blog DS, Rosenber ES. Assessing racial and ethnic disparities using a COVID-19 outcomes continuum for New York State. *Ann Epidemiol*. 2020;48:9-14.

3. Rubin-Miller L, Alban C, Artiga S, Sullivan S. *COVID-19 Racial Disparities in Testing, Infection, Hospitalization, and Death: Analysis of Epic Patient Data*.; 2020. Accessed November 21, 2020. https://www.kff.org/coronavirus-covid-19/issue-brief/covid-19-racial-disparities-testing-infection-hospitalization-death-analysis-epic-patient-data/

4. Chatterjee R. Blacks, Latinos And Native Americans Bear Heaviest Financial Burden Of Pandemic : Shots - Health News : NPR. *National Public Radio*. https://www.npr.org/sections/health-shots/2020/09/18/912731744/how-the-pandemic-is-widening-the-racial-wealth-gap. Published 2020. Accessed November 21, 2020.

5. McNicholas C, Poydock M. Who are essential workers?: A comprehensive look at their wages, demographics, and unionization rates. *Economic Policy Institute*. https://www.epi.org/blog/who-are-essential-workers-a-comprehensive-look-at-their-wages-demographics-and-unionization-rates/. Published May 19, 2020. Accessed November 21, 2020.

6. Centers for Disease Control (CDC). Newly Resettled Refugee Populations | COVID-19. Coronavirus Disease 2019 (COVID-19). Published 2020. Accessed November 21, 2020. https://www.cdc.gov/coronavirus/2019-ncov/need-extra-precautions/refugee-populations.html

7. Panchal N, Kamal R, Orgera K, et al. *The Implications of COVID-19 for Mental Health and Substance Use*.; 2020. Accessed November 19, 2020. https://www.kff.org/coronavirus-covid-19/issue-brief/the-implications-of-covid-19-for-mental-health-and-substance-use/

8. Pfefferbaum B, North CS. Mental Health and the Covid-19 Pandemic. *N Engl J Med*. 2020;383(6):510-512. doi:10.1056/nejmp2008017

9. The Lancet Infectious Diseases. The intersection of COVID-19 and mental health. *Lancet Infect Dis*. 2020;20(11):1217. doi:10.1016/S1473-3099(20)30797-0

10. Alcántara C, Casement MD, Lewis-Fernández R. Conditional risk for PTSD among Latinos: A systematic review of racial/ethnic differences and sociocultural explanations. *Clin Psychol Rev*. 2013;33(1):107-119. doi:10.1016/j.cpr.2012.10.005

11. Garcini LM, Murray KE, Zhou A, Klonoff EA, Myers MG, Elder JP. Mental Health of Undocumented Immigrant Adults in the United States: A Systematic Review of Methodology and Findings. *J Immigr Refug Stud*. 2016;14(1):1-25. doi:10.1080/15562948.2014.998849

12. Sullivan MM, Rehm R. Mental health of undocumented Mexican immigrants: A review of the literature. *Adv Nurs Sci*. 2005;28(3):240-251. doi:10.1097/00012272-200507000-00006

13. Perreira KM, Ornelas I. Painful passages: Traumatic experiences and post-traumatic stress among U.S. immigrant Latino adolescents and their primary caregivers. *Int Migr Rev*. 2013;47(4):976-1005. doi:10.1111/imre.12050

14. Fuligni A, Perreira K. Immigration and Adaptation. In: Villarruel F, Carlo G, Grau J, Azmitia M, Cabrerea N, Chahin T, eds. *Handbook of US Latino Psychology: Developmental and Community-Based Persepctives*. SAGE Publications; 2009.

15. Annan J, Green EP, Brier M. Promoting recovery after war in Northern Uganda: Reducing daily stressors by alleviating poverty. *J Aggress Maltreatment Trauma*. 2013;22(8):849-868. doi:10.1080/10926771.2013.823636

16. Kim I. Beyond Trauma: Post-resettlement Factors and Mental Health Outcomes Among Latino and Asian Refugees in the United States. *J Immigr Minor Heal*. 2016;18:740-748. doi:10.1007/s10903-015-0251-8

17. Miller KE, Rasmussen A. War experiences , daily stressors and mental health five years on : Elaborations and future directions. *Intervention*. 2014;12(1):33-42. doi:10.1097/WTF.0000000000000066

18. Vega WA, Sribney WM, Aguilar-Gaxiola S, Kolody B. 12-month prevalence of DSM-III-R psychiatric disorders among Mexican Americans: nativity, social assimilation, and age determinants. *J Nerv Ment Dis*. 2004;192(8):532-541. doi:10.1097/01.nmd.0000135477.57357.b2

19. Vega W, Kolody B, Valle R, Weir J. Social networks, social support, and their relationship to depression among immigrant Mexican women. *Hum Organ*. 1991;50:154-162.

20. Carrillo J, Trevino F, Betancourt J, Coustasse A. The role of insurance, managed care, and institutional barriers. In: Aguirre-Molina M, Molina C, Zambrano R, eds. *Health Issues in the Latino Community*. Jossey-Bass; 2001.

21. Summary Health Statistics Table for US Populations: National Health Interview Survey, 2015. Published 2017. Accessed January 5, 2017. www.cdc.gov/nchs/fastats/hispanic-health.htm.

22. Lauderdale DS, Wen M, Jacobs E, Kandula N. Immigrant perceptions of discrimination in health care: The California Health Interview Survey 2003. *Med Care*. 2006;44:914-920.

23. Alvarez CP, Davidson PM, Fleming C, Glass NE. Elements of Effective Interventions for Addressing Intimate Partner Violence in Latina Women: A Systematic Review. Carpenter DO, ed. *PLoS One*. 2016;11(8):e0160518. doi:10.1371/journal.pone.0160518

24. Kaltman S, de Mendoza AH, Serrano A, Gonzales FA. A Mental Health Intervention Strategy for Low-Income, Trauma-Exposed Latina Immigrants in Primary Care: A Preliminary Study. *Am J Orthopsychiatry*. 2016;86(3):345-354. doi:10.1037/ort0000157

25. Pierce S, Bolter J. *Dismantling and Reconstructing the U.S. Immigration System: A Catalog of Changes under the Trump Presidency | Migrationpolicy.Org*.; 2020. Accessed November 21, 2020. https://www.migrationpolicy.org/research/us-immigration-system-changes-trump-presidency

26. Ruiz JM, Steffen P, Smith TB. Hispanic mortality paradox: A systematic review and meta-analysis of the longitudinal literature. *Am J Public Health*. 2013;103(3):52-60. doi:10.2105/AJPH.2012.301103

27. Franzini L, Ribble J, Keddie A. Understanding the Hispanic paradox. *Ethn Dis*. 2001;11:496-518.

28. Miranda J, Nakamura R, Bernal G. Including ethnic minorities in mental health intervention research: a practical approach to a long-standing problem. *Cult Med Psychiatry*. 2003;27:467-486.

29. Alegría M, Canino G, Shrout PE, et al. Prevalence of Mental Illness in Immigrant and Non-Immigrant U.S. Latino Groups. *Am J Psychiatry*. 2008;165(3):359-369. doi:10.1176/appi.ajp.2007.07040704

30. Breslau J, Aguilar-Gaxiola S, Kendler K, Su M, Williams D, Kessler RC. Specifying race-ethnic differences in risk for psychiatric disorder in a USA national sample. *Pschological Med*. 2006;36(57-68).

31. Fortuna L, Porche M V., Alegria M. Political violence, psychosocial trauma, and the context of mental health services use among immigrant Latinos in the United States. *Ethn Health*. 2008;13:435-463.

32. Ugalde A, Selva-Sutter E, Castillo C, Paz C, Canas S. Conflict and Health: The health costs of war: Can they be measured? Lessons from El Salvador. *Br Med J*. 2000;321:167-172.

33. Hess J., Parker D, Miramontes I, et al. *Qualitative Analysis of COVID-19 Pandemic Effects on Latinx Immigrants and Positive Impacts of Refugee and Immigrant Well-Being Project*.

34. Trickett EJ, Beehler S, Deutsch C, et al. Advancing the science of community-level interventions. *Am J Public Health*. 2011;101(8):1410-1419. doi:10.2105/AJPH.2010.300113

35. Goodkind, Jessica R.; Hang, P.; Yang M. Hmong Refugees in the United States: A Community-Based Advocacy and Learning Intervention. - PsycNET. In: Miller, Kenneth E.; Rasco LM, ed. *The Mental Health of Refugees: Ecological Approaches to Healing and Adaptation*. Lawrence Erlbaum Associates Publishers.; 2004:295-334. https://psycnet.apa.org/record/2004-14143-009

36. Goodkind, J.R., Githinji, A., & Isakson B. Reducing Health Disparities Experienced by Refugees Resettled in Urban Areas: A Community-Based Transdisciplinary Intervention Model. In: *Converging Disciplines*. Springer; 2011:41-56.

37. Goodkind JR, Amer S, Christian C, et al. Challenges and Innovations in a Community-Based Participatory Randomized Controlled Trial. *Heal Educ Behav*. 2017;44(1):123-130. doi:10.1177/1090198116639243

38. Goodkind JR. Effectiveness of a community-based advocacy and learning program for Hmong refugees. *Am J Community Psychol*. 2005;36(3-4):387-408. doi:10.1007/s10464-005-8633-z

39. Goodkind JR. Promoting Hmong refugees’ well-being through mutual learning: Valuing knowledge, culture, and experience. *Am J Community Psychol*. 2006;37(1):77-93. doi:10.1007/s10464-005-9003-6

40. Goodkind JR, Hess JM, Isakson B, et al. Reducing refugee mental health disparities: A community-based intervention to address postmigration stressors with African adults. *Psychol Serv*. 2014;11(3). doi:10.1037/a0035081

41. Hess JM, Isakson B, Githinji A, et al. Reducing Mental Health Disparities Through Transformative Learning: A Social Change Model With Refugees and Students NIH Public Access. *Psychol Serv*. 2014;11(3):347-356. doi:10.1037/a0035334

42. Goodkind JR, Bybee D, Hess JM, et al. Randomized Controlled Trial of a Multilevel Intervention to Address Social Determinants of Refugee Mental Health. *Am J Community Psychol*. Published online February 17, 2020:ajcp.12418. doi:10.1002/ajcp.12418

43. Vasquez Guzman CE, Hess JM, Casas N, et al. Latinx/@ Immigrant Inclusion Trajectories: Individual Agency, Structural Constraints, and the Role of Community-Based Organizations in Immigrant Mobilities. *Am J Orthopsychiatry*. Published online 2020. doi:10.1037/ort0000507

44. Rusch D, Frazier SL, Atkins M. Building capacity within community-based organizations: new directions for mental health promotion for Latino immigrant families in urban poverty. *Adm Policy Ment Heal Ment Heal Serv Res*. 2015;42(1):1-5.

45. Foster-Fishman PG, Berkowitz SL, Lounsbury DW, Jacobson S, Allen NA. Building Collaborative Capacity in Community Coalitions: A Review and Integrative Framework. *Am J Community Psychol*. 2001;29(2):241-261. doi:10.1023/A:1010378613583

46. Cordero-Guzmán HR. Community-based organisations and migration in New York City. *J Ethn Migr Stud*. 2005;31(5):889-909. doi:10.1080/13691830500177743

47. Reitmanova S, Gustafson DL. Mental health needs of visible minority immigrants in a small Urban Center: Recommendations for policy makers and service providers. *J Immigr Minor Heal*. 2009;11(1):46-56. doi:10.1007/s10903-008-9122-x

48. Viruell-Fuentes E a., Miranda PY, Abdulrahim S. More than culture: Structural racism, intersectionality theory, and immigrant health. *Soc Sci Med*. 2012;75(12):2099-2106. doi:10.1016/j.socscimed.2011.12.037

49. Fosse E, Bull T, Burström B, Fritzell S. Family Policy and Inequalities in Health in Different Welfare States. *Int J Heal Serv*. 2014;44(2):233-253. doi:10.2190/HS.44.2.c

50. Link BG, Phelan J. Social conditions as fundamental causes of disease. *J Heal Soc Behav*. 1995;Spec No:80-94. https://www.ncbi.nlm.nih.gov/pubmed/7560851

51. Navarro V, Shi L. The political context of social inequalities and health. *Soc Sci Med*. 2001;52(3):481-491. doi:10.1016/S0277-9536(00)00197-0

52. Abraido-Lanza AF, Echeverria SE, Florez KR. Latino Immigrants, Acculturation, and Health: Promising New Directions in Research. *Annu Rev Public Heal*. 2016;37:219-236. doi:10.1146/annurev-publhealth-032315-021545

53. Martinez O, Wu E, Sandfort T, et al. Evaluating the Impact of Immigration Policies on Health Status Among Undocumented Immigrants: A Systematic Review. *J Immigr Minor Heal*. 2015;17(3):947-970. doi:10.1007/s10903-013-9968-4

54. Patler C, Laster Pirtle W. From undocumented to lawfully present: Do changes to legal status impact psychological wellbeing among latino immigrant young adults? *Soc Sci Med*. Published online 2017. doi:10.1016/j.socscimed.2017.03.009

55. Hatzenbuehler ML, Prins SJ, Flake M, et al. Immigration policies and mental health morbidity among Latinos: A state-level analysis. *Soc Sci Med*. 2017;174:169-178. doi:10.1016/j.socscimed.2016.11.040

56. Philbin MM, Flake M, Hatzenbuehler ML, Hirsch JS. State-level immigration and immigrant-focused policies as drivers of Latino health disparities in the United States. *Soc Sci Med*. 2018;199:29-38. doi:10.1016/j.socscimed.2017.04.007

57. Link B, Hatzenbuehler ML. Stigma as an Unrecognized Determinant of Population Health: Research and Policy Implications. *J Heal Polit Policy Law*. 2016;41(4):653-673. doi:10.1215/03616878-3620869

58. Ayón C, Becerra D. Mexican Immigrant Families Under Siege: The Impact of Anti-Immigrant Policies, Discrimination, and the Economic Crisis. *Adv Soc Work*. 2013;14(1):206-228.

59. Goodkind J, Hess J, Hernandez-Vallant A, Vasquez Guzman CE, Handal A. From multi-level to trans-level interventions: Creating sustainable social change to improve mental health.

60. Hess J, Goodkind JR, Vasquez Guzman CE, et al. Innovative Participatory Bilingual Data Analysis with Latinx/@ Immigrants: Language, Power, and Transformation. *Cult Divers Ethn Minor Psychol Minor Psychol*.

61. Anderson LM, Adeney KL, Shinn C, Safranek S, Buckner-Brown J, Krause LK. Community coalition-driven interventions to reduce health disparities among racial and ethnic minority populations. *Cochrane Database Syst Rev*. 2015;(6):1-182. doi:10.1002/14651858.CD009905.pub2

62. Cyril S, Smith BJ, Possamai-Inesedy A, Renzaho AM. Exploring the role of community engagement in improving the health of disadvantaged populations: a systematic review. *Glob Heal Action*. 2015;8:1-12. doi:10.3402/gha.v8.29842

63. De Las Nueces D, Hacker K, Digirolamo A, Hicks LS. A systematic review of community-based participatory research to enhance clinical trials in racial and ethnic minority groups. *Health Serv Res*. 2012;47(3 PART 2):1363-1386. doi:10.1111/j.1475-6773.2012.01386.x

64. Drahota A, Meza RD, Brikho B, et al. Community-Academic Partnerships: A Systematic Review of the State of the Literature and Recommendations for Future Research. *Milbank Q*. 2016;94(1):163-214. doi:10.1016/j.ijrobp.2012.07.1399

65. O’Mara-Eves A, Brunton G, Oliver S, Kavanagh J, Jamal F, Thomas J. The effectiveness of community engagement in public health interventions for disadvantaged groups: A meta-analysis. *BMC Public Health*. 2015;15(1):1-24. doi:10.1186/s12889-015-1352-y

66. Salimi Y, Shahandeh K, Malekafzali H, et al. Is Community-based Participatory Research (CBPR) Useful? A Systematic Review on Papers in a Decade. *Int J Prev Med*. 2012;3(6):386-393.

67. Minkler M, Garcia AP, Rubin V, Wallerstein N. *Community Based Participatory Research: A Strategy for Building Healthy Communities and Promoting Health through Policy Change. A Re;Ort to the California Endowment*. PolicyLink; 2012.

68. Cacari-Stone L, Wallerstein N, Garcia AP, Minkler M. The promise of community-based participatory research for health equity: A conceptual model for bridging evidence with policy. *Am J Public Health*. 2014;104(9):1615-1623. doi:10.2105/AJPH.2014.301961

69. Chung B, Ong M, Ettner SL, et al. 12-month outcomes of community engagement versus technical assistance to implement depression collaborative care: a partnered, cluster, randomized, comparative effectiveness trial. *Ann Intern Med*. 2014;161(10 Suppl):S23-34. doi:10.7326/M13-3011

70. Mohler P, Dorer B, de Jong J, Hu M. *Guidelines for Best Practices in Cross-Cultural Surveys*. Fourth Edi. Survey Research Center, Institute for Social Research; 2016. http://ccsg.isr.umich.edu/index.php/chapters/translation-chapter/translation-overview

71. Pan Y, de la Puente M. *Census Bureau Guideline for the Translation of Data Collection Instruments and Supporting Materials: Documentation on How the Guideline Was Developed*. Statistical Research Division, U.S. Bureau of the Census; 2005. https://www.census.gov/srd/papers/pdf/rsm2005-06.pdf

72. Meaklim H, Swieca J, Junge M, et al. The DSM-5 self-rated level 1 cross-cutting symptom measure identifies high levels of coexistent psychiatric symptomatology in patients referred for insomnia treatment. *Nat Sci Sleep*. 2018;10:377-383. doi:10.2147/NSS.S173381

73. Luo M, Guo L, Yu M, Jiang W, Wang H. The psychological and mental impact of coronavirus disease 2019 (COVID-19) on medical staff and general public - A systematic review and meta-analysis. *Psychiatry Res*. 2020;201(January):113190.

74. Penedo F, Cohen L, Bower J, Antoni M. COVID-19: Impact of the Pandemic and HRQOL in Cancer Patients and Survivors. Unpublished questionnaire. Published online 2020.

75. Hamilton CM, Strader LC, Pratt JG, et al. The PhenX Toolkit: get the most from your measures. *Am J Epidemiol*. 2011;174(3):253-260. doi:10.1093/aje/kwr193

76. Sullivan CM, Bybee D. Reducing Violence Using Community-based Advocacy for Women with Abusive Partners. *J Consult Clin Psychol*. 1999;67(43-53).

77. Moser A, Stuck AE, Silliman RA, Ganz PA, Clough-Gorr KM. The eight-item modified Medical Outcomes Study Social Support Survey: Psychometric evaluation showed excellent performance. *J Clin Epidemiol*. 2012;65(10):1107-1116. doi:10.1016/j.jclinepi.2012.04.007

78. Birman D, Trickett EJ, Vinokurov A. Acculturation and Adaptation of Soviet Jewish Refugee Adolescents: Predictors of Adjustment Across Life Domains. *Am J Community Psychol*. 2002;30(5):585-607. doi:10.1023/A:1016323213871

79. Kessler RC, Üstün B. The World Mental Health (WMH) Survey Initiative Version of the World Health Organization (WHO) Composite International Diagnostic Interview (CIDI). *Int J Methods Psychiatr Res*. 2004;13(2):93-121. doi:10.1016/S0002-9149(99)80503-7

80. Robins L, Wing J, Wittchen H, et al. The Composite International Diagnostic Interview. An epidemiologic Instrument suitable for use in conjunction with different diagnostic systems and in different cultures. *Arch Gen Psychiatry1*. 1988;45(12):1069-1077. doi:10.1001/archpsyc.1988.01800360017003. PMID: 2848472

81. Krieger N, Smith K, Naishadham D, Hartman C, Barbeau EM. Experiences of discrimination: Validity and reliability of a self-report measure for population health research on racism and health. *Soc Sci Med*. 2005;61(7):1576-1596. doi:10.1016/j.socscimed.2005.03.006

82. Prinz RJ, Smith EP, Dumas JE, Laughlin JE, White DW, Barron R. Recruitment and retention of participants in prevention trials involving family-based interventions. *Am J Prev Med*. 2001;20(1 Suppl):31-37. https://www.ncbi.nlm.nih.gov/pubmed/11146258

83. Seed M, Juarez M, Alnatour R. Improving recruitment and retention rates in preventive longitudinal research with adolescent mothers. *J Child Adolesc Psychiatr Nurs*. 2009;22(3):150-153. doi:10.1111/j.1744-6171.2009.00193.x

84. Patton MQ, Quinn M. *Qualitative Research and Evaluation Methods*. 2nd ed. Sage Publications; 2002.

85. Luborsky MR, Rubenstein RL. Sampling in qualitative research rationale, issues, and methods. *Res aging* . 1995;17(1):89-113. doi:PMID: 22058580 PMCID: PMC3207270 DOI: 10.1177/0164027595171005

86. Trotter RT, Needle RH, Goosby E, Bates C, Singer MA. A methodological model for rapid assessment, response, and evaluation: The RARE program in public health. *Field methods*. 2001;13:137-159.

87. Goodkind J. *MSU Refugee Well-Being Project Training Manual*. Michigan State University; 2000.

88. Sullivan CM. *Resilient Child Study Advocate Training Manual*. Michigan State University; 1998.

89. Hedeker D, Gibbons RD. Application of random-effects pattern-mixture models for missing data in longitudinal studies. *Psychol Methods*. 1997;2(1):64.

90. Ngaruye I, von Rosen D, Singull M. Small-area estimation with missing data using a multivariate linear random effects model. *Japanese J Stat Data Sci*. 2018;1(1):23-37.

91. Huque MH, Carlin JB, Simpson JA, Lee KJ. A comparison of multiple imputation methods for missing data in longitudinal studies. *BMC Med Res Methodol*. 2018;18(1):168.

92. Grund S, Lüdtke O, Robitzsch A. Multiple imputation of multilevel missing data: An introduction to the R package pan. *Sage Open*. 2016;6(4):2158244016668220.

93. Elkington KS, Bauermeister JA, Zimmerman MA. Psychological distress, substance use, and HIV/STI risk behaviors among youth. *J Youth Adolesc*. 2010;39(5):514-527.

94. Welsh J, Korda RJ, Banks E, Strazdins L, Joshy G, Butterworth P. Identifying long-term psychological distress from single measures: evidence from a nationally representative longitudinal survey of the Australian population. *BMC Med Res Methodol*. 2020;20(1):1-9.

95. Stanton SCE, Selcuk E, Farrell AK, Slatcher RB, Ong AD. Perceived partner responsiveness, daily negative affect reactivity, and all-cause mortality: A 20-year longitudinal study. *Psychosom Med*. 2019;81(1):7.

96. Piazza JR, Stawski RS, Sheffler JL. Age, daily stress processes, and allostatic load: A longitudinal study. *J Aging Health*. 2019;31(9):1671-1691.

97. Chung Y, Maguire-Jack K. Understanding Movement into Poverty and Poverty Persistence over Time. *J Poverty*. 2020;24(3):241-255.

98. Duncan GJ, Rodgers WL. Longitudinal aspects of childhood poverty. *J Marriage Fam*. Published online 1988:1007-1021.

99. Maydeu-Olivares A. Maximum likelihood estimation of structural equation models for continuous data: Standard errors and goodness of fit. *Struct Equ Model A Multidiscip J*. 2017;24(3):383-394.

100. Satorra A, Bentler PM. Corrections to test statistics and standard errors in covariance structure analysis. Published online 1994.

101. Kupzyk KA, Beal SJ. Advanced issues in propensity scores: Longitudinal and missing data. *J Early Adolesc*. 2017;37(1):59-84.

102. Haviland A, Nagin D, Rosenbaum P, et al. Combining group-based trajectory modeling and propensity score matching for causal inferences in nonexperimental longitudinal data. *Health Place*. 2020;39(1):422. Accessed February 25, 2020. https://onlinelibrary.wiley.com/doi/abs/10.1002/ajcp.12418

103. Pals SL, Murray DM, Alfano CM, Shadish WR, Hannan PJ, Baker WL. Individually randomized group treatment trials: a critical appraisal of frequently used design and analytic approaches. *Am J Public Health*. 2008;98(8):1418-1424.

104. Murray DM, Varnell SP, Blitstein JL. Design and analysis of group-randomized trials: a review of recent methodological developments. *Am J Public Health*. 2004;94(3):423-432.

105. MacKinnon DP. *Introduction to Statistical Mediation Analysis (Multivariate Applications Series)*. 1st Editio. Routledge; 2008.

106. Muthen LK, Muthen BO. *MPlus User’s Guide*. Eighth Edi. Muthen & Muthen; 2017.

107. Humphrey SE, LeBreton JM. The handbook of multilevel theory, measurement, and analysis. Published online 2019.

108. Krull JL, MacKinnon DP. Multilevel mediation modeling in group-based intervention studies. *Eval Rev*. 1999;23(4):418-444.

109. MacKinnon DP, Warsi G, Dwyer JH. A simulation study of mediated effect measures. *Multivariate Behav Res*. 1995;30(1):41-62.

110. Preacher KJ, Rucker DD, Hayes AF. Addressing moderated mediation hypotheses: Theory, methods, and prescriptions. *Multivariate Behav Res*. 2007;42(1):185-227.

111. Gustavson K, von Soest T, Karevold E, Røysamb E. Attrition and generalizability in longitudinal studies: findings from a 15-year population-based study and a Monte Carlo simulation study. *BMC Public Health*. 2012;12(1):918.

112. Erdem Ö, Van Lenthe FJ, Burdorf A. Income inequality and psychological distress at neighbourhood and municipality level: An analysis in the Netherlands. *Health Place*. 2019;56:1-8.

113. Erdem Ö, Burdorf A, Van Lenthe FJ. Ethnic inequalities in psychological distress among urban residents in the Netherlands: A moderating role of neighborhood ethnic diversity? *Health Place*. 2017;46:175-182.

114. Eguchi H, Tsutsumi A, Inoue A, Hikichi H, Kawachi I. Association of workplace social capital with psychological distress: results from a longitudinal multilevel analysis of the J-HOPE Study. *BMJ Open*. 2018;8(12).

115. Faul F, Erdfelder E, Buchner A, Lang A-G. Statistical power analyses using G* Power 3.1: Tests for correlation and regression analyses. *Behav Res Methods*. 2009;41(4):1149-1160.

116. Faul F, Erdfelder E, Lang A-G, Buchner A. G* Power 3: A flexible statistical power analysis program for the social, behavioral, and biomedical sciences. *Behav Res Methods*. 2007;39(2):175-191.

117. Charmaz K. *Constructing Grounded Theory*. 2nd ed. SAGE Publications; 2014.

118. Miles MB, Huberman AM, Saldaña J. *Qualitative Data Analysis: A Methods Source Book*. 3rd ed. SAGE Publications Inc.; 2013.

119. Creswell JW PCV. *Designing and Constructing Mixed Method Research.* SAGE Publications; 2011.

120. Creswell JW, Creswell J. *Research Design: Qualitative, Quantitative, and Mixed Methods Approaches*. 5th ed. Sage Publications; 2018.

121. Palinkas L a., Aarons G a., Horwitz S, Chamberlain P, Hurlburt M, Landsverk J. Mixed method designs in implementation research. *Adm Policy Ment Heal Ment Heal Serv Res*. 2011;38(1):44-53. doi:10.1007/s10488-010-0314-z
